# Supplementary material for: Evaluation of an enhanced service for medication review with follow up in Swiss community pharmacies: Pre-post study protocol
Source: PLoS One. 2023 Oct 17;18(10):e0292037. doi: 10.1371/journal.pone.0292037 (PMC10581489; doi:10.1371/journal.pone.0292037)
Supplement: S2 Appendix — (PDF) [file pone.0292037.s002.pdf]

Appendix 2. Eligible patients form (original version in French)

Formulaire d’inclusion des patient.e.s

Identification du/de la patient·e		Critères d'inclusion		Critères d’exclusion							Sélection du/de la patient·e	Inclusion du/de la patient·e	Raison de refus exprimée par le/la patient·e
**Ordre de prise de contact**	Code patient·e	Prise d'au moins 4 médicaments chroniques depuis au moins 3 mois	Patient·e majeur·e	Problème de démence, désordre psychiatrique ou un autre problème de santé empêchant l’ obtention de son consentement éclairé et/ou la réalisation d’ un entretien avec le pharmacien	A déjà bénéficié d’ une réconciliation médicamenteuse dans les six mois précédent son entrée dans l’ étude	Ne parle pas couramment français	Incapacité de déplacer tous ses médicaments	Refuse de s’ entretenir avec le pharmacien dans T0, T6 et T12	Refuse de participer à l’ étude	Refuse la transmission du plan de médication et le rapport des PLM au médecin généraliste	Invité·e à participer à l'étude	Accepte de participer à létude	
**1**													
**2**													
**3**													
**4**													
**5**													
**6**													
**7**													
**8**													
**9**													
**10**													

………

**45**													
**46**													
**47**													
**48**													
**49**													
**50**													
