# Supplementary material for: Evaluation of an enhanced service for medication review with follow up in Swiss community pharmacies: Pre-post study protocol
Source: PLoS One. 2023 Oct 17;18(10):e0292037. doi: 10.1371/journal.pone.0292037 (PMC10581489; doi:10.1371/journal.pone.0292037)
Supplement: S3 Appendix — (PDF) [file pone.0292037.s003.pdf]

Appendix 3. The TIDieR (Template for Intervention Description and Replication) Checklist

TIDieR
Template for Intervention Description and Replication

Information to include when describing an intervention and the location of the information

Number	Item	Where located **
1.	BRIEF NAME	Medication review with follow up (MRF).
2.	WHY	Nowadays, the Swiss "fee for service" convention that set the basis for community pharmacists' remuneration details what pharmacists have to do in order to prevent, identify and manage drug related problems (DRPs) at the time of the delivery of prescription medications to patients (check for drug-drug interactions, contraindications, dosage, etc.). However, there is no service that specifically targets the prevention, identification and management of DRPs possibly induced by the patients' behaviour (adherence, wrong conservation, missing or forgotten information, etc.). The Cantonal Health Authorities and pharmacist association in the canton of Vaud set up a project for the medication review with follow up service (MRF) in collaboration with the Centre for Primary Care and Public Health of the University of Lausanne (Unisanté). The aim of the MRF service is to perform a medication review at the community pharmacy (CP) to: 1) achieve a decrease in the number of DRPs associated to patients' behaviour, 2) improve patients' knowledge about their treatments and 3) remove of expired medications for proper elimination by the CPs. This service allows the pharmacists going further than usual prescription validation in terms of detection of DRPs and pharmaceutical interventions. Based on the principles of the "brown bag" and medication review with follow up, MRF includes all the treatments currently used by the patient (prescription and non-prescription medication), who brings them to the CP before performing the service. MRF is a service that offers several advantages: first, the material aims to guide and facilitate the detection of DRPs through the use of a validated tool and to record

		pharmaceutical interventions as well as offering information to the prescribing general medical practitioner (GP). This service also offers patients a review of self-medication (non-prescription medication). MRF strengthens the role of pharmacy technicians to perform an initial sorting of the medication brought to the CP to eliminate expired drugs, to allow the pharmacist an optimal use of the time devoted to the consultation. The service is in line with the national objective to reinforce pharmacy technicians' role, their education and to allow them to assist (under supervision) some services. The development and implementation of this service is an opportunity for the Centre for Primary Care and Public Health of the University of Lausanne (Unisanté) to conduct a research project.
**3.**	**WHAT**	**MRF:** the service is provided in three stages: before, during and after the pharmacist-patient consultation to review all the medication taken and brought by the patient. Different tasks performed by the pharmacist or pharmacy technicians are also differentiated.<br>To ameliorate the delivery of the intervention, pharmacists will attend a half-day course (four hours) that will cover service provision, good practice standards, writing reports to patients and other health professionals, data collection and study protocol. The training will include a combination of lecture presentations and interactive sessions including role-play scenarios. The training will be accredited for pharmacists' continuing professional development and evaluated according to current standards. After training, pharmacists in each CP will inform and train pharmacy technicians for their tasks using an online version of the training about the service and data collection (two online versions will be available, for pharmacists and pharmacy technicians). There will also have a web page that summarize the information. In addition, participant pharmacists will be followed-up by the research team via telephone during the study at least six times during the study (1st, 2nd, 3rd, 6th, 12th and 15th months of the study) to support them through the delivery of the service. Pharmacists will also be able of contacting the research team by

		email throughout the study concerning service provision, data entry, etc. The facilitation process will be explained to pharmacists during the training session to ensure recruitment targets, quality of service provision and fidelity to study protocol are met.
**4.**	**Procedures**	Before the pharmacist-patient consultation: the purpose of this step is to prepare the necessary documents and the medication for the pharmacist-patient consultation. Patients will be asked to bring all their medication (prescribed and non-prescribed) to the CP. The pharmacy technician will pre-fill the documents with the patient information (medication plan). Pharmacy technicians will also review the medication brought by the patient to divide it in two groups (prescription and non-prescription medication) to identify the prescribed medication currently included in the treatment plan and expired medications for the pharmacist-patient consultation. Then, pharmacist must have at least 30 minutes to review the medication that the patient is currently taking to assess potential DRPs before the consultation.<br>During the pharmacist-patient consultation: the purpose is to systematically identify, using the medication plan, potential DPRs and discuss all DRPs associated to patients' behaviour identified (before and during the consultation) to propose interventions with the patient or the GP to resolve them. For the classification of DRPs, the PharmDISC (including the 15 DRPs related to patients out of the 24 total DRP) will be used; this tool also allows documenting and classifying the pharmaceutical interventions planned by the pharmacist. In addition, during the consultation, the pharmacist will evaluate patients' knowledge using a questionnaire (seven questions) about their medication to improve it, if necessary.<br>After the pharmacist-patient consultation: within a maximum of two working days' period after the consultation with the patient, pharmacists will send a medication plan to both, patient and GP. Additional information will be sent to the GP in case his/her intervention is needed to solve the DRPs detected.

**5.**	**WHO PROVIDED**	Registered pharmacists will provide the MRF after attending a 4-hour training.<br><br>Pharmacy technicians will perform some tasks before the pharmacist-patient consultation.
**6.**	**HOW**	Pharmacists will contact up to 50 randomly selected adult patients who meet inclusion criteria to be included in the study. For those patients who accept to participate, MRF will be provide through three face-to-face encounters between the pharmacist and the patient with six-month intervals, so individual consultations will be carried out in the CP consultation room.
**7.**	**WHERE**	CPs of the canton of Vaud (French part of Switzerland) who voluntarily accept to participate in the study. In order to participate, CPs must have a consultation room and employ at least one pharmacist who will complete the training and will include at least one patient in the study. A CP may have different pharmacists who participate in the study, for those cases, all of them should complete the training and they have to choose a champion pharmacist as a contact person with the research team. A pharmacist who works in different participating pharmacies will be assigned to the CP where he/she works higher number of hours per week.
**8.**	**WHEN and HOW MUCH**	The intervention will be provided by the community pharmacist in three consultations at 6-month intervals alongside the study (15 months). Pharmacy technicians will prepare the documents for the consultation and triage the expired medication.<br>Both, pharmacy technicians and community pharmacists will write on the forms the time needed to complete their tasks to provide the service. Time required for MRF service provision is one of the variables of the study.
**9.**	**TAILORING**	Patients will be eligible if they were adults with a prescription for four or more chronic drugs prescribed for at least three months prior to recruitment. Exclusion criteria includes: patients suffering from dementia, psychiatric disorder, or other health condition that hinders obtaining informed consent and/or conducting the interviews with the pharmacist; patients who received a medication review within the last six months prior their study enrolment; patients who disagree meeting the pharmacist three times during the study with six-month interval; patients who are not able to bring all their medication to the CP; patients who

		cannot speak and read French; patients who does not allow the pharmacist contacting the GP to inform him/her about possible DRPs or patients who will not consent of participating in the study.
**10.**	**MODIFICATIONS**	No changes will be made in the intervention during the study.
**11.**	**HOW WELL (planned)**	MRF is an enhanced service planned by the Cantonal Health Authorities and pharmacist association in the canton of Vaud in collaboration with the Centre for Primary Care and Public Health of the University of Lausanne (Unisanté). The service aims to overcome those difficulties or barriers found in the implementation process of medication review services. The service includes validated, structured and standardized interventions, training and supervision for CP staff, self-medication (non-prescription medication) evaluation and use of home-based patient data (medication).<br>A member of the research team will made phone calls at least six times alongside the duration of the study (1st, 2nd, 3rd, 6th, 12th and 15th months of the study) and will be available by email to assist pharmacists throughout the study.<br>Pharmacists will receive a remuneration of CHF 100 per pharmacist-patient consultation (which cannot be financed by the basic health insurance) and an additional amount of CHF 10 for the additional time spent on the research during each documented consultation according to the guidelines in the educational training. The amount of CHF 100 was calculated by the time expected for each professional involved in the service: approximately 45 minutes for pharmacists (CHF 87 per hour) and approximately 45 minutes for pharmacy technicians (CHF 53 per hour).
**12.**	**HOW WELL (actual)**	The different forms used during the study will be recorded in the pharmacy using an electronic format (Microsoft Excel®) and they will be coded. Study data will be collected and managed using Research Electronic Data Capture (REDCap 12.5.4© 2022 Vanderbilt University) hosted at Unisanté (29) to improve data privacy and data reliability. The research team will ensure that the service is provided as planned (fidelity of the intervention).
