# Supplementary material for: Evaluation of an enhanced service for medication review with follow up in Swiss community pharmacies: Pre-post study protocol
Source: PLoS One. 2023 Oct 17;18(10):e0292037. doi: 10.1371/journal.pone.0292037 (PMC10581489; doi:10.1371/journal.pone.0292037)
Supplement: S4 Appendix — (PDF) [file pone.0292037.s004.pdf]

Appendix 4. Medication plan (original version in French)

Entretien à T 0 **Date entretien:** 16.01.23 | N° Patient·e | 0101

Médicaments prescrits			Dosage	Posologie					Nom du/de la médecin prescripteur·rice (A enlever avant envoi)	Utilisation des médicaments (scores -1=Incorrect ; 0=Ne sait pas ; 1=Correct ;								PLM/ Intervention pharmaceutique
DCI	Nom commercial	Forme galénique		Matin	Midi	Soir	Coucher	Réserve		Indication ?	Quand ?	Combien ?	Repas ?	Oublis ?	Effets indésirables ?	Conservation?	Score par médicaments	
																	0	
																	0	
																	0	
																	0	
																	0	
																	0	
																	0	
																	0	
																	0	
																	0	
																	0	
																	0	
																Score total :	0	
																Score total/nombre de médicaments :	#DIV/0!	

Médicaments non prescrits Nom commercial - forme galénique	Dosage	Mode de prise					Indication rapportée par le/la patient·e	PLM/ Intervention
Le/la patient·e a pris un traitement non prescrits durant les trois derniers mois? Non								

Remarques:
