# Supplementary material for: Evaluation of an enhanced service for medication review with follow up in Swiss community pharmacies: Pre-post study protocol
Source: PLoS One. 2023 Oct 17;18(10):e0292037. doi: 10.1371/journal.pone.0292037 (PMC10581489; doi:10.1371/journal.pone.0292037)
Supplement: S5 Appendix — (PDF) [file pone.0292037.s005.pdf]

Médicaments à Jour? - MaJ? Study of feasibility of a pharmaceutical service for patients with polypharmacy

Research legislation:	Ordinance on human research with the exception of Clinical trials (HRO) [1].
Type of Research Project:	Research project involving human subjects
Risk Categorisation:	Risk category A
Project leader :	PD Dr Jérôme Berger, Pharmacien chef, Centre universitaire de médecine générale et santé publique, Unisanté, Secteur Pharmacie – Recherche. Rue du Bugnon 44, 1011 Lausanne, Suisse / Institut des Sciences Pharmaceutiques de Suisse Occidentale, Université de Genève, Université de Lausanne. Rue Michel Servet 1, 1206 Genève, Suisse. 021 314 48 43 (secrétariat). jerome.berger@unisante.ch.

TABLE OF CONTENTS

GLOSSARY OF ABBREVATIONS

BASEC	*Business Administration System for Ethical Committees*
CP	*Community pharmacies*
CRF	*Case report form*
DGS	*“Direction Générale de la Santé”*
DRP	*Drug related problem*
FOPH	*Federal Office of Public Health*
GP	*General medical practitioners*
HRA	*Human Research Act*
HRO	*Ordinance on Human*
MaJ?	*“Médicaments à Jour ?”*
PCNE	*Pharmaceutical Care Network Europe*
PMC	*PolyMedication Check*
RBP	*“Rémunération Basée sur les Prestation”*
SVPh	*“Société Vaudoise de Pharmacie”*

1 BACKGROUND AND PROJECT RATIONALE

1.1 Role of the pharmacist

The role of the community pharmacist includes an increasing number of responsibilities. Whereas in the past the aspects related to the manufacture and sale of medicines were predominant (1), the 2000s saw the emergence of other roles for the pharmacist in Switzerland, primarily in order to control rising health care costs (2,3). With the implementation of the “Rémunération Basée sur les Prestation” (RBP) in 2001, a step forward was taken in the direction of pharmaceutical services, for example by the remuneration of the pharmaceutical record during the delivery of prescribed medicines (4). Since 2001, the RBP has been amended several times in order to respond to the health care system needs and the activities of community pharmacists in relation to patients’ needs (adherence, polymedication, etc.) (5).

According to the Federal Council, pharmacists could still take on additional tasks in the field of primary care, as they offer easier access to health advice (6). Since 2016, the “Grand Conseil du Canton de Vaud” has been supporting the implementation of a shared medication plan concerning "medication safety throughout the care process". The aim is to evaluate the implementation of a collaborative management of patients’ treatment based on the e-health platform for the canton of Vaud on polymedicated patients with multiple carers (general medical practitioners –GP-, community pharmacies -CP- and nursing homes) (7). In this context, new opportunities exist to strengthen the role of the pharmacist in the care process. In addition to prescribed medications, pharmacists also advise patients when they self-medicate. Self-medication is "the selection and use of medication, including herbal and traditional products, by individuals to treat symptoms or diseases that they have identified themselves" (8). Community pharmacists are often the first health professionals to be contacted by patients with minor ailments (9,10). In order to ensure responsible self-medication with non-prescription medication, pharmacist advice is necessary (11).

Therefore, community pharmacists are in a privileged position to have an overview of all the medicines taken by patients (prescription and non-prescription) (12,13). A study carried out in the Canton of Vaud in 2017 with diabetic patients showed that 79% of them had visited only one pharmacy in the last 12 months (96% two pharmacies maximum) (14).

1.2 Drug-related problems (DRPs)

The Pharmaceutical Care Network Europe (PCNE) describes a DRP as "an event or circumstance involving drug therapy that actually or potentially interferes with the expected optimal outcome of medical care" (15). The PCNE has developed a classification of DRPs that has evolved over time and among investigators. Thus, the variation in classifications makes it difficult to standardize this topic, especially on a more accurate description of the clinical and economic burden of DRP (16,17).

As an example, it has been estimated that the costs of DRP such as non-adherence is approximately 125 billion euro per year in the European Union (18). In Switzerland, each year, 20,000 hospitalizations result from DRP (6). DRPs have several causes, whether related to the patient or to the health process (15). For example, if the patient is not familiar with his/her treatment, he/she will not be motivated to take it, especially if the disease is chronic and the symptoms are not obvious (19-21). In this situation, an adherence problem could arise resulting in health care costs (hospitalizations, medical visits, etc.) that could have been avoided (11).

Another example of DRP could be a treatment intake error (inadequate posology, etc.) due to a drug package that is misunderstood or missing or due to old medication storage at home. Intake errors could lead to a decrease of treatment effectiveness or to an increase in adverse effects and risks of drug interactions (22).

Another example of DRP could be inadequate storage of medicaments. Treatments such as eye drops could lead to eye infections if they are not properly storage (23). Similarly, improperly stored insulin pens could lose their effectiveness due to a decrease in insulin concentration (24).

These sources of DRP can be prevented and managed through pharmaceutical interventions (25). A pharmaceutical intervention is described as “any action performed by the pharmacist that result in a change in treatment or therapy management” (26). It is well known that pharmaceutical interventions, such as patient counseling, can reduce DRP (27,28).

One way to identify and prevent the occurrence of DRP in a structured manner is to conduct a medication review with the patient (29).

To document community pharmacists’ clinical activities, including the identification and management of DRPs, a tool was developed and validated in Swiss CPs (30).

1.3 Medication Review

Medication review is defined as "a structured evaluation of the patient's medications with the goal of optimizing their use and improving health status", it involves the detection of DRPs and the recommendation of interventions (31, 32).

Nowadays, different review models have been studied in many countries (33). However, few of them have been evaluated in CP.

Medication review in pharmacy

Brown Bag

First described in 1982, this type of medication review was originated in the United States (34). Patients were invited to bring all their medications stored at home to the CP for a review of prescribed and non-prescribed medications by the pharmacist (35). The presence of allergies or intolerances was also investigated (35). This type of review allows an effective identification of DRP and encourages patients to discuss their treatment with their GPs (35). This service considers all the medications stored in the patient's home, not only the contents of the pharmaceutical record, which may sometimes be incomplete (36). However, the effectiveness of this review method depends on the willingness of patients to bring back all their medications, as well as on a systematic review by the pharmacist (37).

Medication review in Switzerland

In 2010, a new service was added to the RBP (38), called PolyMedication Check (PMC). Through this service, the pharmacist can improve the patient's knowledge about treatments and, if necessary, suggest ways of improving adherence (39). The service enables a drug review to be carried out by combining two sources of information: the contents of the pharmaceutical record and the consultation with the patient. The PMC does not include a clinical drug review.

The PMC is effective in identifying DRPs; however, its effectiveness in reducing the number of DRPs, has not been demonstrated (29).

DRPs detected and the pharmaceutical interventions performed during the PMC are not systematically communicated to the prescribing GP (29). Thus, PMC has not shown to enhance interprofessional collaboration. However, as this is a tool recognized by the Swiss health insurance available to pharmacists to facilitate drug review, it is important to optimize it in order to obtain a new service that is not only useful for the identification of DRPs, but that also allows them to be managed more effectively.

"Médicaments à Jour?” Medication review to be integrated into the shared medication plan

In 2017 the Société Vaudoise de Pharmacie (SVPh) set up the project "Médicaments à Jour?" (MaJ?), in collaboration with Unisanté Pharmacy and the “Direction Générale de la Santé” (DGS) in the canton of Vaud. Based on the PMC, this service allows the pharmacists going further in terms of detection of DRP and pharmaceutical interventions. Based on the principles of the Brown Bag, MaJ? includes all the treatments consumed by the patient, who brings them back to the CP to perform a medication review.

MaJ? is a service that offers innovations over the PMC: first, the material related to the PMC service has been optimized to facilitate the detection of DRP and to record pharmaceutical interventions as well as offering information to the prescribing GP. This service also offers patients the opportunity of disposing unused medications at the CP (40), which increases patients’

education about proper disposal of medications to reduce their ecological impact (41). Second, an assessment of patient self-medication is also performed. Medications that are not part of the medication plan prescribed by the GP, are discussed with the patient and included in the medication record to be shared with GPs and patients.

Differently to PMC, MaJ? also includes the role of pharmacy technicians to perform an initial sorting of the medication brought to the CP by the patient. It facilitates the elimination of expired or unused drugs, to allow the pharmacist an optimal use of the time devoted to the consultation.

An initial pilot study of 51 patients recruited from 13 pharmacies was carried out (42).

1.4 Risk category

The present project comes under Category A as there is no sampling for biological material and the burdens or risks while collecting data is minimal for the patients. The interviews carried out by the pharmacists with patients do not present risks to patients and allows to structure and document clinical activities usually conducted in community pharmacies. If needed, when the pharmacist's interventions involve prescription medication, the intervention could be first discussed with the patient's general medical practitioner according to the usual way of coordinating clinical activities between professionals.

2 PROJECT OBJECTIVES AND DESIGN

2.1 Hypothesis and primary objective

MaJ? will allow a better identification and management of DRPs in community pharmacy in addition to structuring and documenting this clinical activity.

2.2 Primary and secondary endpoints

Primary objective:
To evaluate the impact of MaJ? on the identification and management of DRP.
Secondary objectives:
To assess the impact of MaJ? on the number of expired or untaken medications.
To assess the impact of MaJ? on patients' knowledge about their treatments.
To describe the interventions made by the pharmacists through MaJ?.

2.3 Project design

Pre-post intervention study carried out in Swiss CPs from the canton of Vaud (multicenter). Each CP will be considered a cluster to avoid contamination. The study will take place over 15 months between April 2023 and June 2024.

3 PROJECT POPULATION AND STUDY PROCEDURES

3.1 Project population, inclusion and exclusion criteria

In order to participate in this study, patients must meet the following inclusion criteria:

- Patients with a prescription for at least four chronic drugs for at least the last three months.
- Adult patients, 18 years old or older.

Patients meeting one or more of the following exclusion criteria will not be included in the study:

- Patients suffering from dementia, psychiatric disorder, or other health condition that hinders obtaining informed consent and/or conducting the consultations with the pharmacist.

- Patients receiving a PMC within the last six months prior their study enrollment.

- Patients who disagree meeting the pharmacist for the first consultation (T0), second consultation six months later (T6) or third consultation twelve months after the first one (T12).

- Patients who are not able to bring all their medication to the CP.

- Patients who cannot speak and read French.

- Patients who does not allow the pharmacist contacting the general medical practitioner (GP) to inform him/her about possible DRPs.

- Patients who will not consent of participating in the study.

Primary objective of the study aims to measure the difference in DRPs per patient observed between T0-T6, and T0-T12. Sample size calculation is based on this primary study outcome to detect a difference of 0.5 DRP per patient at the end of the study. The sample size was calculated with ≥0.8 power, type I error rate of 5%, assuming an intra-cluster correlation of 0.02. Allowing for 15% dropout, the overall sample size is 162 patients, with 19 to 35 pharmacies (1-10 patients per pharmacy).

3.2 Recruitment, screening and informed consent procedure

The Pharmaceutical Association of Vaud (SVPh) and the DGS will provide CPs with the study information via email. In order to participate in this study, CPs must meet the following criteria:

- Employ at least one pharmacist who has completed the MaJ? training to perform the service.

- Name a pharmacist as the contact pharmacist, who agrees to represent the pharmacy for communication purposes with the research team and for submitting study data within a specified period (T0, T6 and T12).

- To include at least one patient in the study.

In order to participate in this study, pharmacists must meet the following criteria:

- Participate in MaJ? training session.

A pharmacist may care for multiple patients but a patient may speak at T0, T6 and T12 with the same trained pharmacists. A pharmacist who works in different participating pharmacies will be assigned to the pharmacy where he/she works higher number of hours per week. After initial training, new eligible pharmacists who start working in participant pharmacies will be able to participate if complete the online training (see Appendix 1).

Pharmacists will receive a remuneration of CHF 100 per consultation (which cannot be financed by the basic health insurance) and an additional amount of CHF 10 for the additional time spent on the research during each documented consultation according to the guidelines in the educational training.

The CP will send the list of patients in the pharmacy with the inclusion criteria (this list will not include patients' identification but CP codes) for which they will also receive a compensation of CHF 100. In order to avoid selection bias during recruitment, a member of the research team will randomize 50 patients (CP codes) from the list (randomized through a sequence of computer-generated random numbers). The research team will send an inclusion form to the contact pharmacist of each CP with the 50 patients randomized for them to contact the patients by phone or in person in CP. Pharmacist will evaluate that patients do not meet exclusion criteria (see Appendix 2) and they will ask patients for their oral informed consent (see Appendix 3) tracking record of the oral consents (see Appendix 2). Participating pharmacists will be asked to enroll at least one to ten patients and they will use advertisements/flyers to inform patients about the service provide at the CP (see Appendix 4). Patients will have ample time for consideration about their inclusion in the study with the opportunity to ask questions to the pharmacists and they will not receive payment to participate in the study.

Figure 1. Processus de sélection des patients

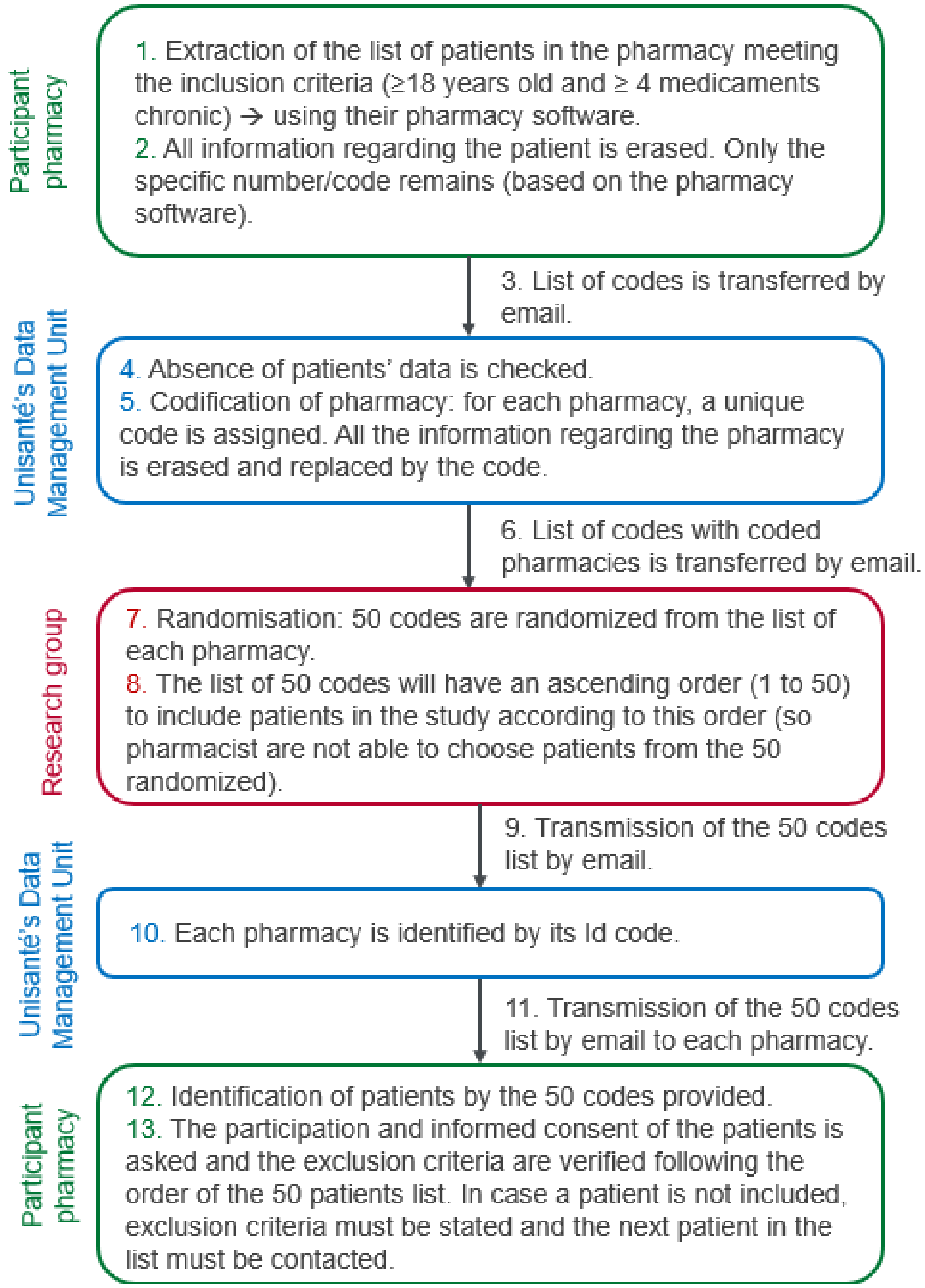

3.3 Study procedures

3.3.1 Pharmacy staff training

Educational training for pharmacists will be divided in two trainings with a duration of two hours each one. The first part will be carried out online and the second part will be carried out in person in the Unisanté premises. It will cover service provision, good practice standards, patients' recruitment, communication skills with patients and other health professionals, data collection and study protocol (Appendix 1). Training will be recognize as postgraduate and continue education for community pharmacists (FPH). Pharmacists will inform pharmacy

technicians of their tasks, as in usual practice they will bear the responsibility for these tasks, and they will also have available a specific online version of the training about the service and data collection for pharmacy technicians.
During patient enrollment, pharmacists will be contacted by the research team to keep them informed of recruitment progress and to motivate them to enroll new patients. They will be follow-up by the research team during the study at least six times (1st, 2nd, 3rd, 6th, 12th and 15th months of the study) to support them through the delivery of the service. These contacts will be made by phone and email. The facilitation process will be explained to pharmacists during the training session. Facilitation process allows ensuring recruitment targets are met, quality of service provision and fidelity to study protocol.

3.3.2 The pharmacy service “Médicaments à Jour?”
The aim of the service is to perform a medication review at the CP to achieve the following goals:
- To decrease the number of DRP.
- To remove expired or untaken medications.
- To improve patients’ knowledge about their treatments.
An intervention plan (sent to the GP) will be developed based on the pharmacy record, treatments reported by the patient and the consultation. Patients will be asked to bring all their medication to the pharmacy, which will be sorted by the pharmacy technician. The medication management plan will be then reviewed and explained to the patient by the pharmacist. The pharmacist will also check to identify potential DRPs such as adherence issues and propose interventions to solve them. Then, the service is provided in three stages to the patient (before, during and after the pharmacist-patient consultation at the CP):

1. Before the pharmacist-patient consultation
The purpose of this step is to prepare the necessary documents for the service before the appointment with the patient. The pharmacy technician will pre-fill the documents with the following information:
Medication management plan (Appendix 5):
- Patient Id: pharmacy software codes.
- Medication data: treatment information (name of drug prescribed, dose, posology).
- Medication knowledge evaluation questionnaire: it contains seven questions that will be ask for each medication (Why do you take this medication; When do you take this medication?; How many tablets/pushes/milliliters do you take per dose; How do you take this medicine with food; How do you take this medicine with food; What do you do if you forget to take a dose of this medicine; What side effects may occur with this medicine?; At home, where do you keep this medicine?)
Removed medication form (Appendix 6):
- Patient Id: pharmacy software codes.
- Time spent by the pharmacy technician and pharmacist to provide the service.
- Treatments to be removed: brand name/DCI, dose, formulation.
- Medication by group: an initial medication review by the pharmacy technician will be carried out. This can be accomplished by the patient bringing their own medications prior to the consultation or throughout other arrangements with the pharmacy. The pharmacy technician separates the patient's medications into two groups: Group I (prescribed-medication that are part of the medication plan), Group II (medication outside the medication plan, this could be over the counter-OTC drugs or medication not currently prescribed).
- Pre-triage of medications: made by the patients at home.
- Missing dosage labels: in order to identify drug-related problems related to the absence of a label.

2. During the pharmacist-patient consultation
On the day of pharmacist-patient consultation, the pharmacist will have at least 30 minutes to review the patient's medications before the patient’s arrival. The purpose of this review is to

have the opportunity to assess potential DRP and to list them using the validated and published PharmDISC tool adapted to document DRPs related to patients in the Swiss context of community pharmacy (Appendix 7).
The purpose of this consultation will be to discuss the potential DRP and to evaluate and improve patient's knowledge of his/her treatment using the patient’s medication knowledge evaluation questionnaire. If necessary, the pharmacist will propose different interventions to the patient to resolve identified DRPs which will be recorded using the PharmDISC tool. Patient's self-medication will be discussed during this encounter to resolve patients’ questions. Pharmacists will confirm with the patients the medication to be removed. The pharmacist will also give advice about the disposal of expired or unused medications. Pharmacists will be able of adding comments to each form.

3. After the pharmacist-patient consultation, the pharmacist will to inform the GP by providing report of the consultation with the patient using the updated medication management plan (Appendix 8). In case of different GPs prescribing treatments to the patient, this report may improve communication between health care practitioners and patient’s management. The medication management plan will be also handle to the patient. However, in case of discrepancies between the medication record and the medication brought in by the patient, the pharmacist will forward the form to the GP for validation before handing it to the patient.

Time frame for the intervention (Table 1)
The study will be carried out for 15 months where pharmacists will carry out three different consultations with each patient, at the beginning for the first trimester (T0), six months later (T6) and 12 months after the first encounter (T12). The variables collected at each moment are detailed in table 2 and 3.
The first pharmacist-patient consultation (T0) will take place after patient recruitment. After codifying the data, contact pharmacists in each pharmacy will send a copy of the information to the research team (within one month after the first consultation) by RedCap®.
The second pharmacist-patient consultation will take place 6 months (T6) after the first consultation (T0). The intervention will be conducted similarly to the first consultation and using the same forms (including pharmaceutical interventions at T0 and T6). Copy of data will be provided to the research team within one month after completing the second consultation.
The third pharmacist-patient consultation will take place 12 months (T12) after T0. The intervention will be conducted similarly to the first (T0) and second (T6) consultation and using the same forms (including pharmaceutical interventions at T0, T6 and T12). Copy of data will be provided to the research team within one month after completing the last consultation.

Table 1. Time frame and process for the intervention

	Before T0	T0	T6	T12
**Timeframe (visits)**	First month of the study	During the first trimester of the study	Six months after the first consultation	Twelve months after the first consultation
**Process**	Check inclusion/exclusion criteria<br>Oral informed consent	Patient brings the medication to the CP (before consultation).<br>Pharmacist-patient consultation (during consultation)<br>Pharmacist prepare reports for patients and GP (after consultation)	Idem to T0	Idem to T0 and T6
**Main variables**	Inclusion and exclusion criteria (appendix 2)<br>Oral consent (appendix 3)<br>Pharmacists-related characteristics (table 3)<br>Pharmacies-related characteristics (table 3)	Patient related characteristics (table 3)<br>Patient medication list (appendix 5)<br>Patient's medication knowledge (appendix 5)<br>Removed medication (expired or unused) (appendix 6)<br>Drug related problems (appendix 7)<br>Pharmacist's interventions (appendix 7)	Idem to T0	Idem to T0 and T6

CP: community pharmacy; GP: general medical practitioner

Figure 2. Flux des données collectées

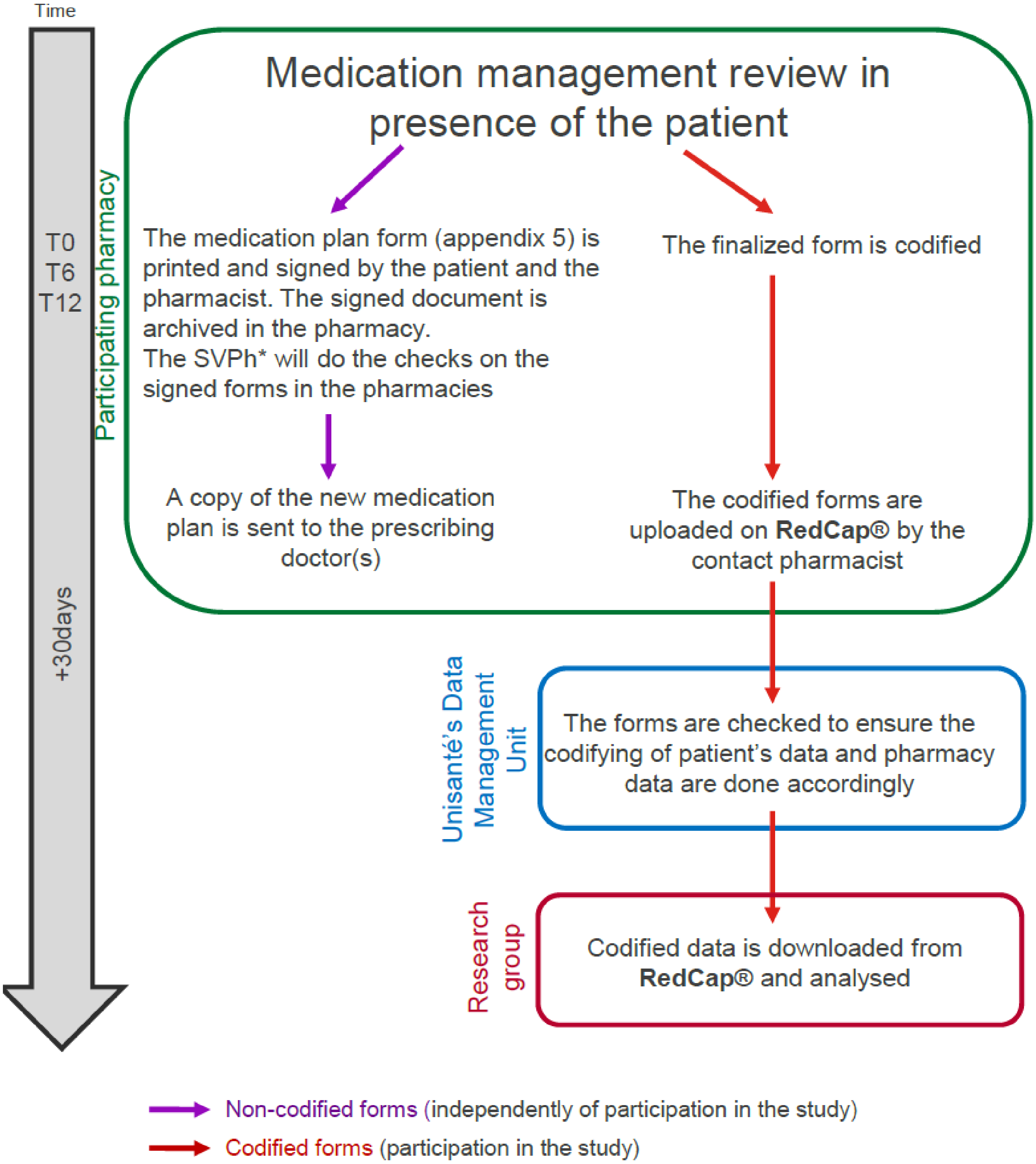

*SVPh: Société Vaudois de Pharmacie

Table 2. Study dependent variables

Outcomes and variables	Variable type	Definition and assessment	Time point
**Participation**	Quantitative	In order to test the interest and feasibility of the service, participation rate will be calculated as the number of patients who accepted to participate in the study of those who were proposed to participate in the study after randomization.	Pharmacists and pharmacy technicians will complete an inclusion document at the beginning of the study, listing all patients who accept to participate.
**Service duration**	Quantitative (minutes)	Time invested by the pharmacists and the pharmacy technicians to deliver the intervention (recorded separately). Time invested at T0, T6 and T12 and total duration will be considered.	Pharmacy technicians (before the consultation) and pharmacists (after the consultation) will complete the medication management plan at T0, T6 and T12.
**Number of medications propose to be removed**	Quantitative	Medication expired will be identified after reviewing the medication brought by the patient to the pharmacy. Total number, mean per patient and percentage in relation to the total medication brought by the patient will be documented.	Pharmacy technicians will complete the medication management plan before the pharmacist-patient consultation at T0, T6 and T12.
**Number of medications removed**	Quantitative	Number of medication removed accepted by the patient after pharmacist-patient consultation.	Pharmacists will complete the medication management plan during the consultation at T0, T6 and T12.
**Patient's knowledge**	Quantitative	Medication knowledge evaluation questionnaire with 7 questions (Why do you take this medication; When do you take this medication?; How many tablets/pushes/milliliters do you take per dose; How do you take this medicine with food; How do you take this medicine with food; What do you do if you forget to take a dose of this medicine; What side effects may occur with this medicine?; At home, where do you keep this medicine?). The patient scores 1 point if he/she knows the correct answer, 0 points if	Pharmacists will complete the questionnaire with the patient during the consultation at T0, T6 and T12.

		he/she does not know and -1 if the information given is incorrect. Score goes from -7 (patient does not know the medication) to 7 (patient completely knows the medication) for each medication that the patient is taking.<br>The questionnaire is being validated nowadays in a separate project.<br>The total score and the mean score (total score divided by the number of medicines) will be documented.	
**Number of DRPs detected**	Quantitative	DRPs detected by the pharmacist before the consultation (evaluation of treatments) and during the consultation (evaluation of patients' information). Total number of DRPs and mean per patient will be calculated.	Pharmacists will complete the medication management plan and PharmDISC tool before and during the consultation at T0, T6 and T12.
**DRP classification**	Qualitative	PharmDISC tool (Appendix 7) will be used for DRP classification:<br>1. Therapy choice: duplication, adverse effect.<br>2. Dose choice: under dose/overdose.<br>3. Drug use: inappropriate timing of frequency of administration, inappropriate use method, inappropriate therapy duration.<br>4. Patient: insufficient adherence, insufficient knowledge, burden due to therapy, treatment costs, incorrect storage, absence of lable.<br>5. Logistic: prescribed drug not available, error in medication process.<br>6. Other.	Pharmacists will complete the PharmDISC tool before and after the consultation at T0, T6 and T12.
**Number of pharmaceutical interventions**	Quantitative	Number of interventions carried out by the pharmacist.<br>Total number of interventions and mean per pharmacist and patient will be calculated.	Pharmacists will complete the PharmDISC tool before and during the consultation at T0, T6 and T12.
**Pharmaceutical interventions**	Qualitative	Classification of interventions will be made using PharmDISC tool (Appendix 7):<br>- Counselling of patient	Pharmacists will complete the PharmDISC tool before and during at T0, T6 and T12.

		- Application instruction (training)<br>- Delivery of an adherence aid inclusive counselling<br>- Therapy started<br>- Therapy stopped/no delivery<br>- Substitution<br>- Dose adjustment<br>- Therapy monitoring<br>- Optimization of administration<br>- Change of administration way<br>- Adjustment of the delivery amount (package size, quantity of packages, etc.)<br>- Information to the general medical practitioner<br>- Clarification in the case notes (history)<br>- Report to pharmacovigilance center<br>- Other	
**Pharmaceutical interventions acceptance**	Qualitative	Pharmaceutical interventions acceptance by the patient or the general medical practitioner (when proposed to the general medical practitioner, it will be considered as accepted unless stated otherwise).	Pharmacists will complete the PharmDISC tool during the consultation at T6 and T12.
**Pharmacist's opinion**	Qualitative	Pharmacists' opinion with training and MaJ?.<br>Pharmacists' opinion about MaJ? will be measured using a questionnaire submitted (Appendix 9) (8 questions using a likert scale from "Totally disagree" to "Completely agree").	Pharmacists will complete the evaluation questionnaires after the first pharmacist-patient consultation and at the end of the study.
**Evolution on the number of DRPs**	Quantitative	Evolution on the number of DRPs detected will be assessed by comparing the differences between T6-T0 and T12-T0.	Research team will calculate the differences using the medication management plan at T0, T6 and T12.

**DRP severity**	Qualitative	Severity categorization of DRPs following SCOPE criteria. Severity will be classified as mild (I, II), moderate (III, IV) and severe (V and VI).	Research team using the PharmDISC tool completed by the pharmacist at T0, T6 and T12.

Table 3. Study independent variables

**Patient-related**	Socio-demographic characteristics	Gender (man / woman / non-binary / Other)<br>Age interval (18-29 years old / 30-39 years old / 40-49 years old / etc.)
	Treatment	Brand name/DCI<br>Dose<br>Formulation<br>Dosage<br>Posology (if available)<br>Number of prescribers treating the patient<br>Patients receiving a pillbox
**Pharmacists-related**	Socio-demographic characteristics	Gender (man / woman / non-binary / Other)
	Academic and work-related characteristics	Role (adjoin / non–owner manager / owner manager, non-available)<br>Year of graduation in pharmacy<br>Place of graduation in pharmacy (Switzerland / abroad / non-available)<br>Working hours in the pharmacy
**Pharmacies-related**	Prescriptions	Number of prescriptions filled per day
	Pharmacy characteristics	Number of hours the pharmacy is open per week<br>Location of the pharmacy (city / suburb / industrial area / countryside/ non-available)<br>Number of pharmacy technicians employed<br>Number of pharmacists employed<br>Equivalent of full-time pharmacists employed<br>Number of full-time pharmacists participating in the study

3.4 Withdrawal and discontinuation

Eligible patients (determined by being adult, having four or more medications and the patient's agreement) should still have inclusion criteria, identified at recruitment, at the time of the first pharmacist-patient consultation. If, after T0, a patient have any treatment change and inclusion criteria is no longer met (less than four medications), he/she will be able to finish the study to receive the service (T6 and T12).

A patient could withdrawal from the project due to withdrawal of informed consent, change of the community pharmacy (for a pharmacy not involved in the project, i.e. out of the Canton), absence to pharmacist-patient consultations, or death. In case of withdrawal of informed consent, data will be destroyed (by identifying patient's Id), therefore patient's data will not be used in the analysis and study results.

4 STATISTICS AND METHODOLOGY

4.1. Statistical analysis plan

Continuous variables will be reported using mean and standard deviation, or median and percentiles depending on the distribution of the variable (the Kolmogorov Smirnov test will be used to assess normality). Categorical variables will be reported using frequency and proportion. For the comparison of continuous variables, the T Student's test or the ANOVA test will be carried out if there is a normal distribution, and Kruskal-Wallis otherwise. The comparison of the categorical variables will be carried out using the $\chi 2$ test, the Fisher's exact test or Yate's chi-squared test if necessary.

For each patient under study, differences T12-T0 and T6-T0 will be compared for dependent variables (DRP, pharmaceutical interventions, pharmaceutical interventions acceptance, patient knowledge, medication removed). A linear regression model will be performed accounting for the cluster effect. Firstly, variables will be considered significant (p-value<0.2) in a bivariate model to be included in a multivariate model. Secondly, the variables considered significant at a value of $p<0.1$ in the multivariate model will be included in the final model. The level of significance will be set at $p<0.05$, the software STATA® will be used.

4.2. Handling of missing data

Data analysis will be done on an intention-to-treat (ITT) basis, so all patients and pharmacists recruited will be included in the final analyses (unless withdrawal of informed consent). For the management of missing data, initial value will be considered as not been modified (baseline observation carried forward-BOCF) (45).

5 REGULATORY ASPECTS AND SAFETY

5.1 Local regulations / Declaration of Helsinki

This research project will be conducted in accordance with the protocol, the Declaration of Helsinki (46), the principles of Good Clinical Practice, the Human Research Act (HRA) (47) and the Human Research Ordinance (HRO) (48) as well as other locally relevant regulations. The project leader acknowledges his responsibilities as both the project leader and the Sponsor.

5.2 Notification of safety and protective measures (HRA Art. 15, HRO Art. 20)

If, during the research project, circumstances arise which could jeopardise the safety or health of the participants or lead to a disproportionate relationship between the risks and burdens and the benefits, all the measures required to ensure protection are to be taken without delay. The project leader will be promptly notified (within 24 hours) if immediate safety and protective measures have to be taken during the conduct of the research project. The Ethics Committee will be notified via BASEC of these measures and of the circumstances necessitating them within 7 days.

5.3 Serious events (HRO Art. 21)

If a serious event occurs, the research project will be interrupted and the Ethics Committee notified on the circumstances via BASEC within 7 days according to HRO Art. 21 (48).

5.4 Procedure for investigations involving radiation sources

Non applicable.

5.5 Amendments

Substantial changes to the project set-up, the protocol and relevant project documents will be submitted to the Ethics Committee for approval according to HRO Art. 18 before implementation. Exceptions are measures that have to be taken immediately in order to protect the participants.

5.6 End of project

Upon project completion or discontinuation, the Ethics Committee is notified within 90 days. All health-related data will be codified in the pharmacies, therefore, research team will not be able to identify patients.

5.7 Insurance

In the event of project-related damage or injuries, the Sponsor will be liable, except for damages that are only slight and temporary; and for which the extent of the damage is no greater than would be expected in the current state of scientific knowledge (Art. 12 HRO).

6 FURTHER ASPECTS

6.1 Overall ethical considerations

This project will evaluate a service financed by the DGS and the SVPh which allows structuring and documenting activities carried out routinely by CPs: medication review and sorting of patients' medications. The aim of the project is to evaluate whether this service is relevant in order to implement it in the Canton of Vaud and other cantons. The service is considered to improve the medication management from patients and to support the appropriate disposal of medicines.

The effort required from patients is small: they must bring their medication to the CP and spend time with the pharmacist in order to benefit from structured explanations about their treatments. There is no particular clinical risk for patients, in fact, they could benefit from the service outside the study as part of the campaign led by the DGS and SVPh.

Pharmacists are paid for the time spent, so no extra resources are used on other activities detriment.

6.2 Risk-Benefit Assessment

The service provided in the present study is considered to fall under usual practice of community pharmacists. Pharmacists will be able to continue to provide routine care to patients about DRP management. Patients will benefit of having interviews with the pharmacist to further evaluate the medication and medication knowledge in order to improve it. No special medical supervision is required. Pharmacists are free to contact general medical practitioners in agreement with patients as usual.

6.3 Rationale for the inclusion of vulnerable participants

Non applicable.

7 QUALITY CONTROL AND DATA PROTECTION

7.1 Quality measures

Participant pharmacists will be follow-up via telephone during the study at least six times (1st, 2nd, 3rd, 6th, 12th and 15th months of the study) to support them through the delivery of the service. Pharmacists will also be able of contacting the research team by email throughout the study concerning service provision, data entry, etc. The facilitation process will be explained to pharmacists during the training session. Facilitation process allows ensuring recruitment targets are met, quality of service provision and fidelity to study protocol.

For quality assurance the Ethics Committee may visit the research sites. Direct access to the source data and all project related files and documents must be granted on such occasions.

7.2 Data recording and source data

Participating pharmacists will be responsible for keeping all documents in the pharmacy according to current practices and requirements.
The different forms used during the study (medication management plan, removed medication form and PharmDisc tool) will be recorded in the pharmacy using an electronic format (Microsoft Excel®) and they will be collected coded via RedCap® to improve data privacy and data reliability. Data will be double checked by an external data processing agency ("Unité de Gestion de Données de Unisanté) to assure codification is being done correctly by pharmacists. Afterwards, data will be accessible by the research team at Unisanté via RedCap®.
Data will only be accessible by the researchers involved in the project and will be hosted on Unisanté's server (backed up electronically).
At the end of the study, data will be kept up to 10 years after the last publication.

7.3 Confidentiality and coding

Project data will be handled with uttermost discretion and is only accessible to authorized personnel who require the data to fulfil their duties within the scope of the research project. On the CRFs and other project specific documents, participants are only identified by a unique participant number.
Pharmacists will use patients Id already assigned in their pharmacy software (internal codes) to identify patients. An external data processing agency ("Unité de Gestion de Données de Unisanté") will have the list for the pharmacies Ids and it will assure codification of pharmacies is being done correctly by pharmacists before the research team can access data.
Data will only be accessible by the researchers involved in the project and will be hosted on Unisanté's server. The server is only access by personal password that is renewed each six months and its data are backed up electronically.

7.4 Retention and destruction of project data and biological material

No biological material is included in the study. In relation to project's data, it will be kept up to 10 years after the last publication.

8 FUNDING / PUBLICATION / DECLARATION OF INTEREST

There is no contract or any written agreement between the institutions (pharmacies) and the research team. Nevertheless, public-private partnership DGS-SVPh will provide for a reimbursement of CHF 100 per pharmacist-patient consultation and an additional amount of CHF 10 for the additional time spent on the research during each documented consultation according to the guidelines in the educational training. The pharmacists will send the list of patients in the pharmacy with the inclusion criteria (this list will not include patients' identification but CP codes) for which they will also receive a compensation of CHF 100.

There is no compensation for patients included. However, they will benefit of additional services for free.

No conflicts of interest are declared. However, the investigators (pharmacists and research team) are committed to the development of services within their professional competencies. They are committed to handle data in a rigorous and transparent manner to avoid bias in the collecting, interpretation and discussion of the results.

The findings obtained after the study will be disseminated through the usual methods of scientific dissemination, including communications to congresses and publications in scientific journals.

9 REFERENCES

1. Hepler CD, Strand LM. Opportunities and responsibilities in pharmaceutical care. Am J Hosp Pharm. 1990;47(3):533-43.
2. Guignard E, Bugnon O. Pharmaceutical Care in Community Pharmacies: Practice and Research in Switzerland. Ann Pharmacother. 2006;40(3):512-7.
3. Hersberger KE, Messerli M. Development of Clinical Pharmacy in Switzerland: Involvement of Community Pharmacists in Care for Older Patients. Drugs Aging. 2016;33(3):205-11.
4. Pharmasuisse. Convention tarifaire Rémunération Basée sur les Prestations (RBP) IV/1 [Internet]. Pharmasuisse. 2016. [cited 23 February 2022]. Available in: https://www.pharmasuisse.org/data/docs/fr/4711/Convention-tarifaire-RBP-IV-1.pdf?v=1.0
5. Mesnil M. Système de rémunération du pharmacien [Internet]. Université de Genève.2019 [cited 23 February 2022]. Available in: https://chamilo.unige.ch/home/courses/14H052/document/14H052_C19_Le_pharmacien,_acteur_en_politique_de_sante/Annee_academique_2018-2019/14H052_C19_Remuneration_du_pharmacien_-_28-11-2018.pdf?cidReq=14H052&id_session=0&gidReq=0
6. Fondation Sécurité des patients Suisse. Une médication sûre lors de l'admission à l'hôpital [Internet]. Sécurité des patients Suisse. 2019 [cited 23 February 2022]. Available in: https://www.securitedespatients.ch/programmes-pilotes/la-securite-de-la-medication-aux-interfaces/#c1677
7. État de Vaud. Exposé des motifs et projet de décret sur le développement d'outils et de processus favorisant la continuité et la coordination des soins [Internet]. Canton de Vaud. 2016 [cited 23 February 2022]. Available in: https://www.vd.ch/toutes-les-autorites/grand-conseil/seances-precedentes/annee-2016/seance-du-mardi-6-et-mercredi-7-decembre-2016/expose-des-motifs-et-projet-de-decret-sur-le-developpement-doutils-et-de-processus-favorisant-la-continuite-et-la-coordination-des-soins/

8. Rutter P. Role of community pharmacists in patients' self-care and self-medication. Integr Pharm Res Pract. 2015;4:57-65. doi: 10.2147/IPRP.S70403. PMID: 29354520; PMCID: PMC5741028.
9. Pharmasuisse. Moniteur-des-pharmacies-2017. L'essentiel-en-bref [Internet]. Pharmasuisse. 2017 [cited 23 February 2022]. Available in: https://www.pharmasuisse.org/data/docs/fr/7393/Moniteur-des-pharmacies-2017-L%E2%80%99essentiel-en-bref.pdf?v=1.0
10. Pharmasuisse. Faits-et-chiffres-2019 [Internet]. Pharmasuisse. 2017 [cited 23 February 2022]. Available in: https://www.pharmasuisse.org/data/docs/fr/19076/Faits-et-chiffres-2019.pdf?v=1.0
11. Cooper RJ. Over-the-counter medicine abuse – a review of the literature. J Subst Use. 2013;18(2):82-107.
12. Maes KA, Hersberger KE, Lampert ML. Pharmaceutical interventions on prescribed medicines in community pharmacies: focus on patient-reported problems. Int J Clin Pharm. 2018;40(2):335-40.
13. Hamada N. Documentation des problèmes liés aux médicaments (PLM) en pratiques officinale et institutionnelle. [Genève]: Université de Genève et Polyclinique médicale Universitaire; 2015.
14. Bawab N, Berger J. Cohorte des patient·e·s diabétiques du canton de Vaud - Résultats 2017. Université de Genève et Polyclinique médicale Universitaire; 2017.
15. Pharmaceutical Care Network Europe Association (PCNE). Classification for drug related problems V9.1 [Internet]. PCNE. 2020 [cited 23 February 2022]. Available in: https://www.pcne.org/upload/files/417_PCNE_classification_V9-1_final.pdf
16. Krähenbühl J-M, Kremer B, Guignard B, Bugnon O. Practical evaluation of the drug-related problem management process in Swiss community pharmacies. Pharm World Sci. 2008;30(6):777-86.
17. Lim X, Yeo Q, Kng G, Chung W, Yap K. Validation of a Drug-Related Problem Classification System for the Intermediate and Long-Term Care Setting in Singapore. Pharmacy. 2018;6(4):109.
18. Vrijens B, Antoniou S, Burnier M, de la Sierra A, Volpe M. Current Situation of Medication Adherence in Hypertension. Front Pharmacol. 2017;8:100.
19. Linck C de L, Bielemann V de LM, Sousa AS de, Lange C. The chronic patient in face of falling ill and the treatment compliance. Acta Paul Enferm. 2008;21(2):317-22.
20. Rohrer R. Je suis une personne qui crée des liens. Astrea Pharmacie. Healthcare Consulting Group SA. 2018;41-3.
21. Osterberg L, Blaschke T. Adherence to Medication. N Engl J Med. 2005;353(5):487-97.
22. Pérez-Jover V, Mira J, Carratala-Munuera C, Gil-Guillen V, Basora J, López-Pineda A, et al. Inappropriate Use of Medication by Elderly, Polymedicated, or Multipathological Patients with Chronic Diseases. Int J Environ Res Public Health. 2018;15(2):310.
23. Desmeules J. Date de péremption et stabilité des médicaments. Pharma-Flash. 2003;30(6):21-4.
24. Mériot C. L'insuline et ses différents modes d'injection. Actual Pharm. 2008;47(478):12-5.
25. Confédération Suisse. RS 811.11 Loi fédérale du 23 juin 2006 sur les professions médicales universitaires [Internet]. Fedlex. 2021 [cited 23 February 2022]. Available in: https://www.admin.ch/opc/fr/classified-compilation/20040265/index.html
26. Dooley MJ, Allen KM, Doecke CJ, Galbraith KJ, Taylor GR, Bright J, et al. A prospective multicentre study of pharmacist initiated changes to drug therapy and patient management in acute care government funded hospitals: Pharmacist interventions in hospitalized patients. Br J Clin Pharmacol. 2004;57(4):513-21.
27. Hanlon JT, Lindblad CI, Gray SL. Can clinical pharmacy services have a positive impact on drug-related problems and health outcomes in community-based older adults? 2004;3-13.
28. Tasaka Y, Tanaka A, Yasunaga D, Asakawa T, Araki H, Tanaka M. Potential drug-related problems detected by routine pharmaceutical interventions: safety and economic

contributions made by hospital pharmacists in Japan. J Pharm Health Care Sci. 2018;4:33. doi: 10.1186/s40780-018-0125-z.
29. Messerli M, Blozik E, Vriends N, Hersberger KE. Impact of a community pharmacist-led medication review on medicines use in patients on polypharmacy--a prospective randomised controlled trial. BMC Health Serv Res. 2016;16:145.
30. Hamada N, Quintana Bárcena P, Maes KA, Bugnon O, Berger J. Clinical Pharmacy Activities Documented (ClinPhADoc): Development, Reliability and Acceptability of a Documentation Tool for Community Pharmacists. Pharmacy (Basel). 2019;7(4):162. doi: 10.3390/pharmacy7040162.
31. Stewart AL, Lynch KJ. Medication discrepancies despite pharmacist led medication review: the challenges of maintaining an accurate medication list in primary care. Pharm Pract. 2014;12(1).
32. Griese-Mammen N, Hersberger KE, Messerli M, Leikola S, Horvat N, van Mil JWF, et al. PCNE definition of medication review: reaching agreement. Int J Clin Pharm. 2018;40(5):1199-208.
33. Redmond P, Grimes TC, McDonnell R, Boland F, Hughes C, Fahey T. Impact of medication review for improving transitions of care. Cochrane Database Syst Rev. 2018;8(8):CD010791. doi: 10.1002/14651858.CD010791.pub2.
34. Nathan A, Goodyer L, Lovejoy A, Rashid A. « Brown bag » medication reviews as a means of optimizing patients' use of medication and of identifying potential clinical problems. Fam Pract. juin 1999;16(3):278-82.
35. O'Connell MB, Chang F, Tocco A, Mills ME, Hwang JM, Garwood CL, et al. Drug-Related-Problem Outcomes and Program Satisfaction from a Comprehensive Brown Bag Medication Review. J Am Geriatr Soc. 2015;63(9):1900-5.
36. Dersch-Mills D, Hugel K, Nystrom M. Completeness of information sources used to prepare best possible medication histories for pediatric patients. Can J Hosp Pharm. 2011;64(1):10-5.
37. Sarzynski EM, Luz CC, Rios-Bedoya CF, Zhou S. Considerations for using the «brown bag» strategy to reconcile medications during routine outpatient office visits. Qual Prim Care. 2014;22(4):177-87.
38. Messerli M, Vriends N, Hersberger KE. Humanistic outcomes and patient acceptance of the pharmacist-led medication review «Polymedication Check» in primary care in Switzerland: a prospective randomized controlled trial. Patient Prefer Adherence. 2018;12:1071-8.
39. Mediyinal Tarif kommission (MTK). Ambulante tarife sav tarifstruktur 2010 [Internet]. MTK. 2010 [cited 23 February 2022]. Available in: https://www.mtk-ctm.ch/fileadmin/user_upload/tarife/Apotheker-Tarif/02_franzoesisch/ambulante_tarife_sav_tarifstruktur_2010_fra.pdf
40. Confédération Suisse. Elimination des déchets médicaux [Internet]. Office fédéral de l'environnement OFEV. 2004. [cited 23 February 2022]. Available in: https://www.bafu.admin.ch/bafu/fr/home/themes/dechets/publications-etudes/publications/elimination-des-dechets-medicaux.html
41. Ouellet N, Dubé P-A, Pharm B, Tox CC. Retour des médicaments périmés ou inutilisés aux fins de destruction du point de vue de la santé publique. Bull D'information Toxicol. 2014;30(2):47-65.
42. Reitzel C, Bachmann T, Ochsenbein L, Berger J. Médicaments À Jour ? Etude pilote observationnelle. Lausanne; 2018.
43. Formulaire-Entretien-de-polymédication.pdf [Internet]. [cité 16 avr 2019]. Disponible sur: https://members.pharmasuisse.org/data/docs/fr/3991/Formulaire-Entretien-de-polym%C3%A9dication.pdf?v=1.0
44. Quintana-Bárcena P, Lord A, Lizotte A, Berbiche D, Jouini G, Lalonde L. Development and validation of criteria for classifying severity of drug-related problems in chronic kidney disease: A community pharmacy perspective. Am J Health Syst Pharm. 2015;72(21):1876-84.
45. Liu-Seifert H, Zhang S, D'Souza D, Skljarevski V. A closer look at the baseline-observation-carried-forward (BOCF). Patient Prefer Adherence. 2010;4:11-6. doi: 10.2147/ppa.s8135.

46. Declaration of Helsinki (https://www.wma.net/policies-post/wma-declaration-of-helsinki-ethical-principles-for-medical-research-involving-human-subjects)
47. Human Research Act (HRA). http://www.admin.ch/opc/en/classified-compilation/20121176/201401010000/810.305.pdf
48. Ordinance on Human Research with the Exception of Clinical trials (HRO) https://www.admin.ch/opc/en/classified-compilation/20121177/index.html

Appendix 1: Training for pharmacists

«Médicaments A Jour?» - MAJ? Formation en ligne

Mathilde Escaith
Dre Noelia Amador-Fernández
Dr PD Jérôme Berger

Structure de la formation

Formation en ligne

1. Introduction à la prestation
2. Etapes de la prestation
3. Projet de recherche: brève introduction
4. Bonnes pratiques de documentation de la prestation

Formation en présentiel (10.10.22 et 14.11.22)

1. Bonnes pratiques de documentation de la prestation: cas pratique
2. Projet de recherche: transmission des documents

Introduction à la prestation et au projet de recherche

Introduction à la prestation

- Problèmes Liés aux Médicaments (PLM)
- Revue de médication
- MAJ: Qu’est-ce que c’est?
- MAJ: Pour qui?
- PLM visés
- Brève explication du projet de recherche

PLM: définition

Pharmaceutical Care Network Europe (PCNE):

«A Drug-Related Problem is an event or circumstance involving drug therapy that actually or potentially interferes with desired health outcomes»

Définitions

Revue de médication

PCNE: *«Evaluation structurée des traitements d'un·e patient·e afin d'optimiser son traitements et d'améliorer son état de santé. Cela implique la détection de problèmes liés aux médicaments et la recommandation d'interventions»*

Réconciliation

«L'évaluation compréhensive de la médication d'un patient à chaque changement dans la pharmacothérapie dans un effort visant à éviter les erreurs de médication telles que les omissions ou les doublons, ainsi que pour observer l'adhésion médicamenteuse »

PLM: causes et détection

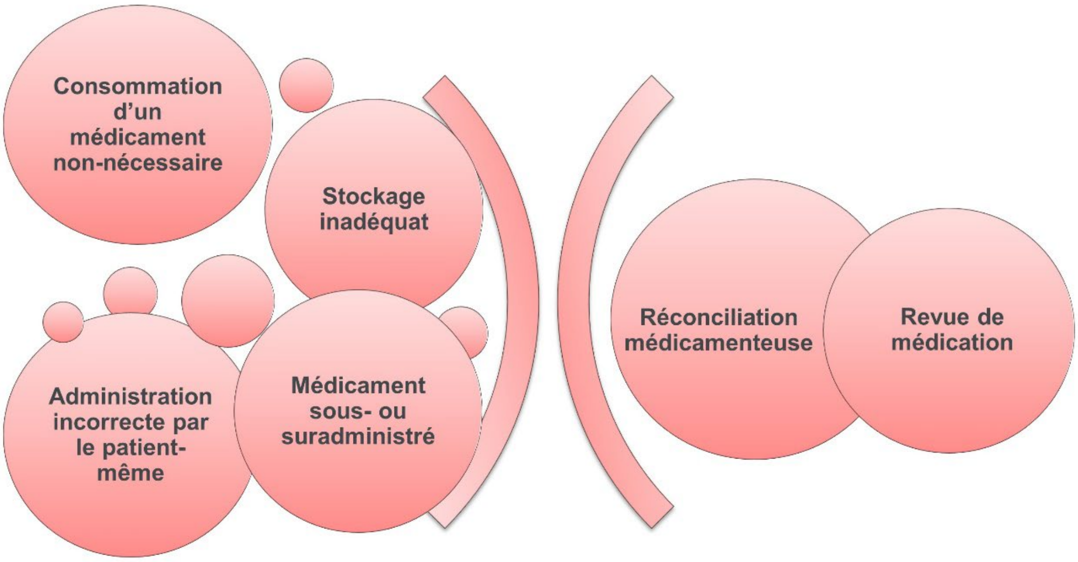

Causes de Problèmes Liés aux Médicaments (PLM) attribuées au patient et au circuit du médicament

Stratégies pour une détection opportune des PLM

Problèmes Liés aux Médicaments visés

- Périmé
- Etiquette de posologie absente
- Posologie incorrecte sur l'étiquette
- Dosage incorrect
- Doublon
- Médicament absent
- Problème d'adhésion
- Stockage inapproprié
- Accumulation
- Ancien traitement prescrit

→ **Il s'agit avant tout d'une revue de l'utilisation des médicaments**

MAJ: objectifs principaux

- Avoir un plan actualisé des médicaments que le/la patient·e prend effectivement (médicaments prescrits et non-prescrits)
- Outil pour l'évaluation de la gestion et des connaissances des médicaments
  → Structuration des entretiens et des interventions
- Avoir un outil de documentation des PLM et des interventions pharmaceutiques faites

MAJ: Pour qui?

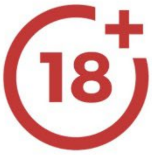

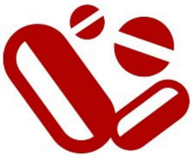

- Patient·e·s **majeur·e·s polymédiqué·e·s**:
  - ≥ 4 médicaments et pris en charge par la caisse maladie
  - Depuis ≥ 3 mois, en vue d'une prise chronique

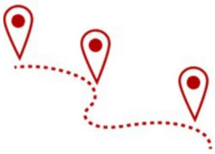

- Capables de se **déplacer** à la pharmacie avec tous leurs médicaments aux 3 entretiens (T0, T6, T12)

MAJ: Qu'est-ce que c'est? (1/2)

- **Réconciliation** médicamenteuse en pharmacie
  - Avec <u>tous</u> les médicaments du/de la patient·e qu'il/elle a à son domicile = médicaments prescrits <u>et</u> non-prescrits (hors plan de médication)
- **Revue de médication** se centrant sur **l'utilisation et la gestion à domicile des médicaments** => détection de **PLM** liés au/à la patient·e et à la gestion de la médication
- Documentation des **PLM et des interventions pharmaceutiques**
- **Élimination** des médicaments échus ou non utilisés
- Plan de **médication** réconcilié et transmis au/à la médecin et au/à la patient·e

MAJ: Qu'est-ce que c'est? (2/2)

<u>Prestation sur rendez-vous</u>: le/la patient·e doit venir avec tous ses emballages de médicament à la pharmacie et une revue des emballages doit être faite avant l'entretien

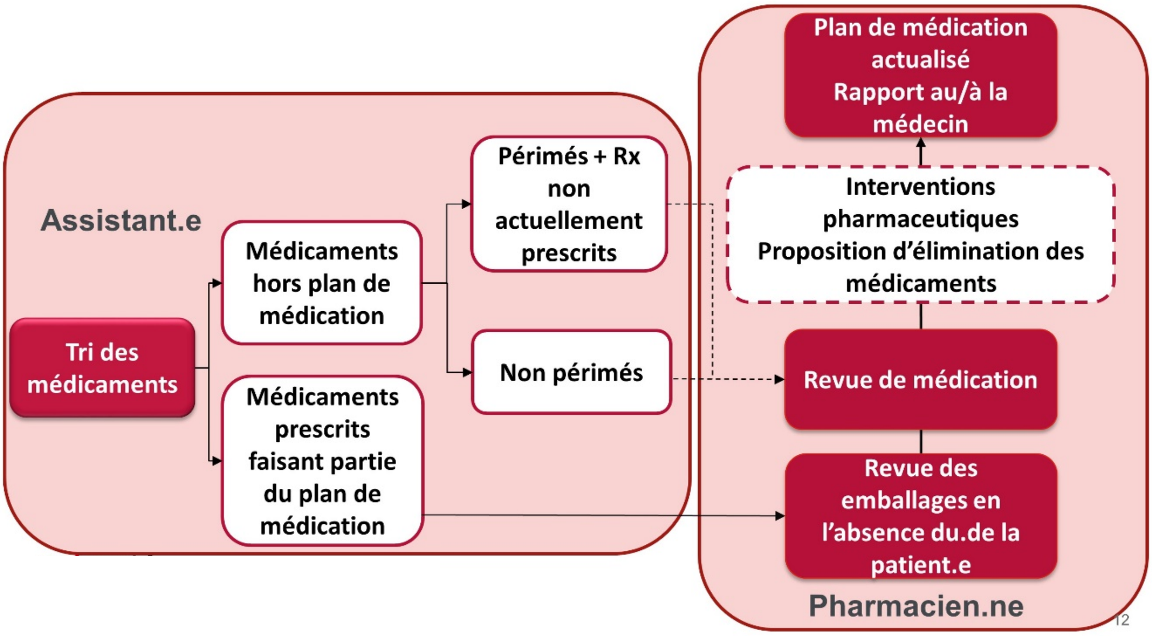

Etapes de la prestation

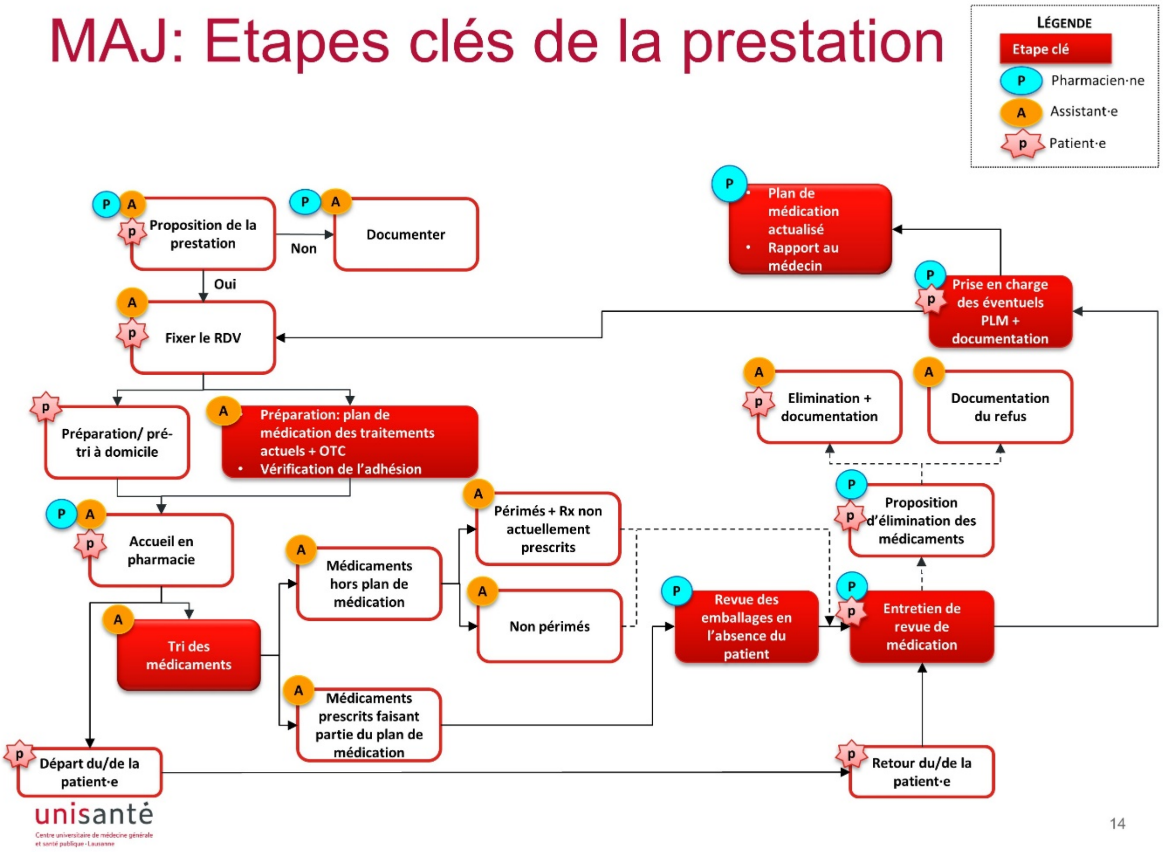

Etape clé : préparation des documents

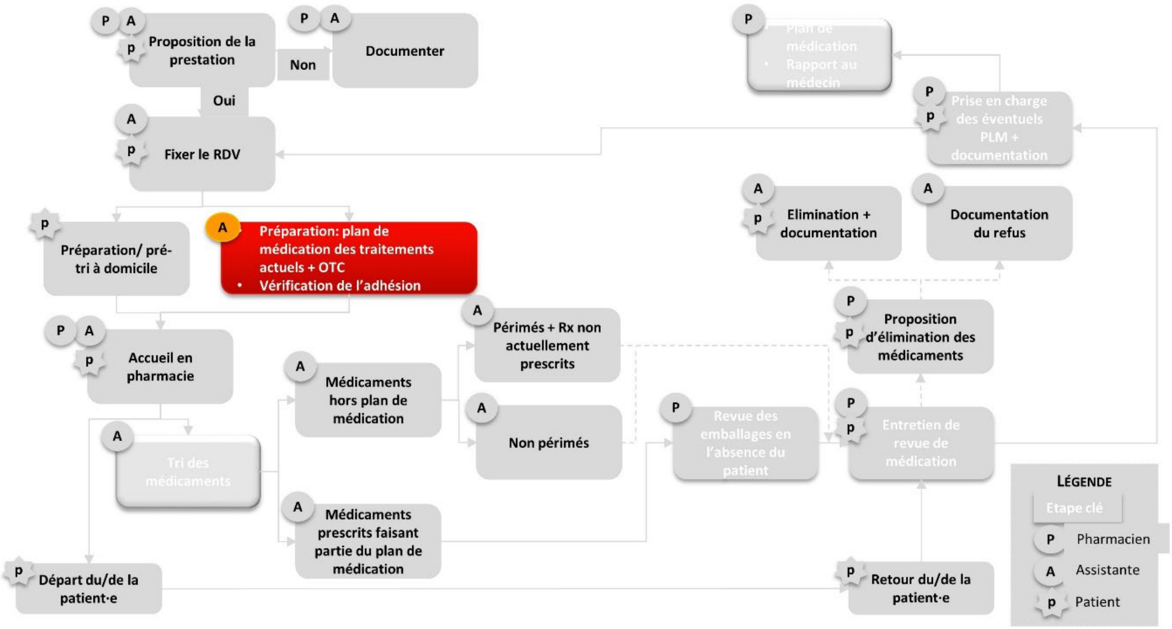

Avant: Préparation des documents

- **Qui** : Assistant·e en pharmacie
- **Source d'information** : dossier pharmaceutique
- **But** : documents prêts pour le RDV. Facilite le tri des médicaments, l'entretien avec le/la patient·e et la rédaction du plan de médication.

Etape clé: Tri des médicaments

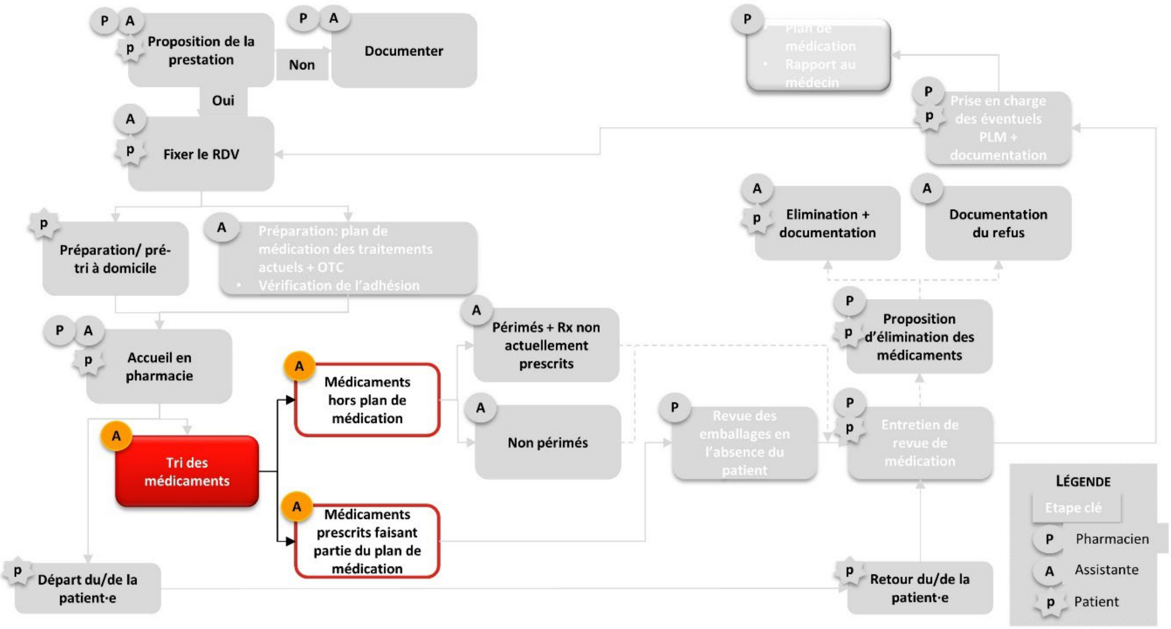

Pendant: Tri des médicaments

- **Qui**: Assistant.e en pharmacie
- **Sources d'information**: dossier pharmaceutique et médicaments ramenés
- **But** : séparer et trier les médicaments ramenés par le/la patient·e:
  - Médicaments **prescrits actuellement** (seront revus plus en détail) et médicaments **non-prescrits actuellement** (OTC, Liste B+, anciens traitements, etc.) pour l'actualisation
  - Détecter **d'éventuels PLM** (date d'expiration, emballages avec des traces d'humidité, etc.)
  - Eliminer des **potentiels biais d'analyse**: pré-tri fait en amont de la venue à la pharmacie? L'absence de posologie est volontaire?

Etapes clés: Revue des emballages

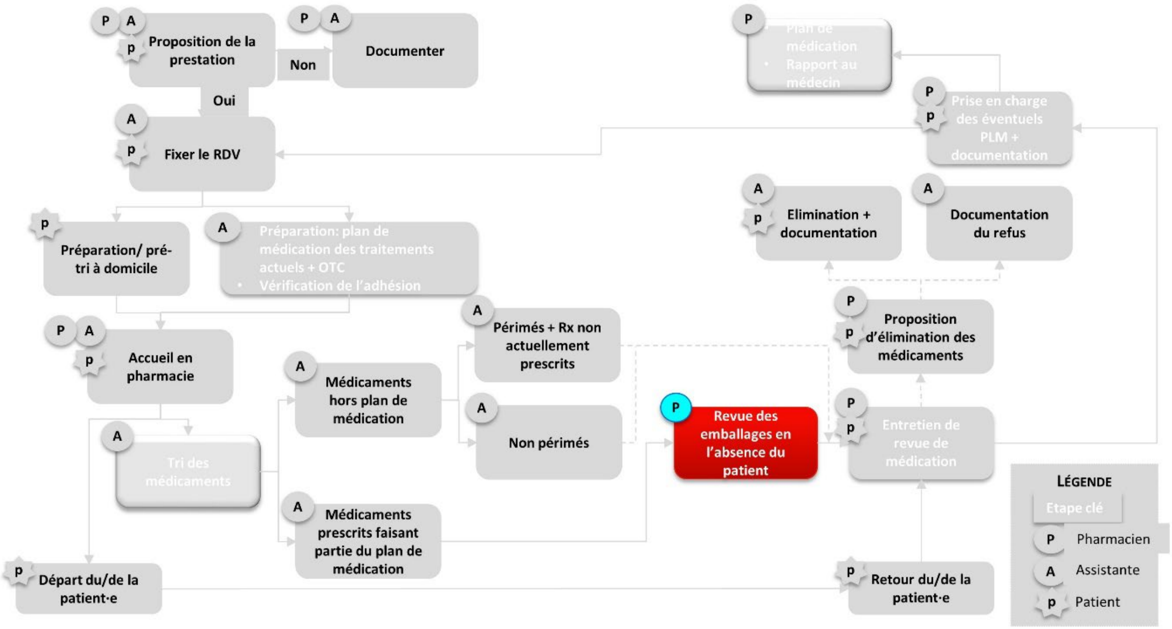

Pendant: Revue des emballages

- **Qui** : Pharmacien.ne
- **Sources d'information** : médicaments triés par l'assistante
- **But** : détecter d'éventuels PLM et préparer l'entretien avec le/la patient·e

NB: L'assistant·e en pharmacie documente les PLM des médicaments **actuellement non-prescrits** lors du tri des médicaments et des potentiels biais d'analyse (pré-tri effectué à domicile, posologies volontairement absentes), le/la pharmacien·ne en prends connaissance et éventuellement complète.

Etape clé: Entretien de réconciliation

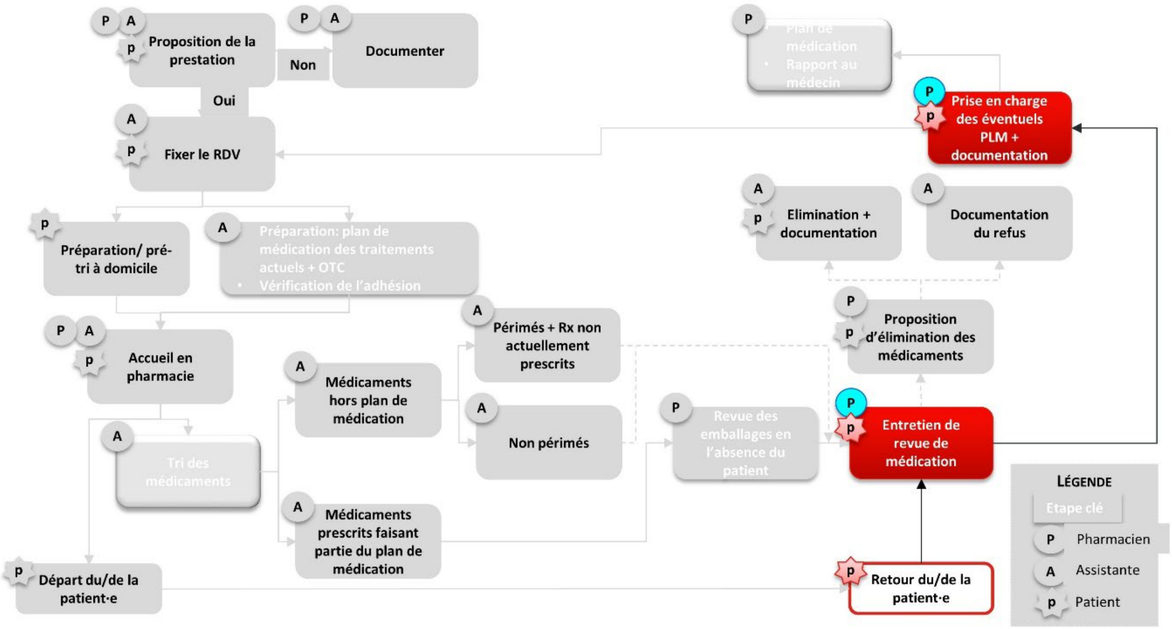

Pendant: Entretien de revue de médication

- **Qui** : Pharmacien·ne
- **Sources d'information** : médicaments triés, observations faites par le/la pharmacien·ne et entretien
- **But** : identifier et gérer avec le/la patient·e et/ou le/la médecin/soignant·e les éventuels PLM des médicaments prescrits

Etape de la prestation: Elimination

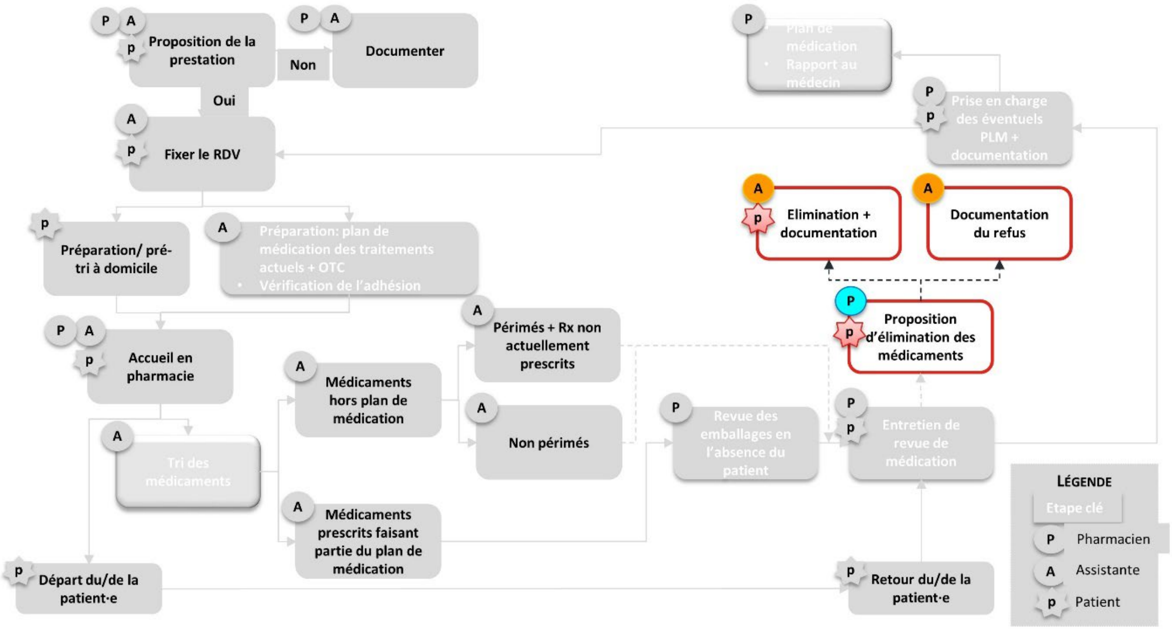

Pendant: Elimination médicaments

- **Qui** : Pharmacien.ne
- **But** : éliminer les médicaments périmés ou non-utilisés pour éviter leur mauvaise utilisation et s'assurer de leur destruction appropriée.

Etape clé: Plan médication et rapport

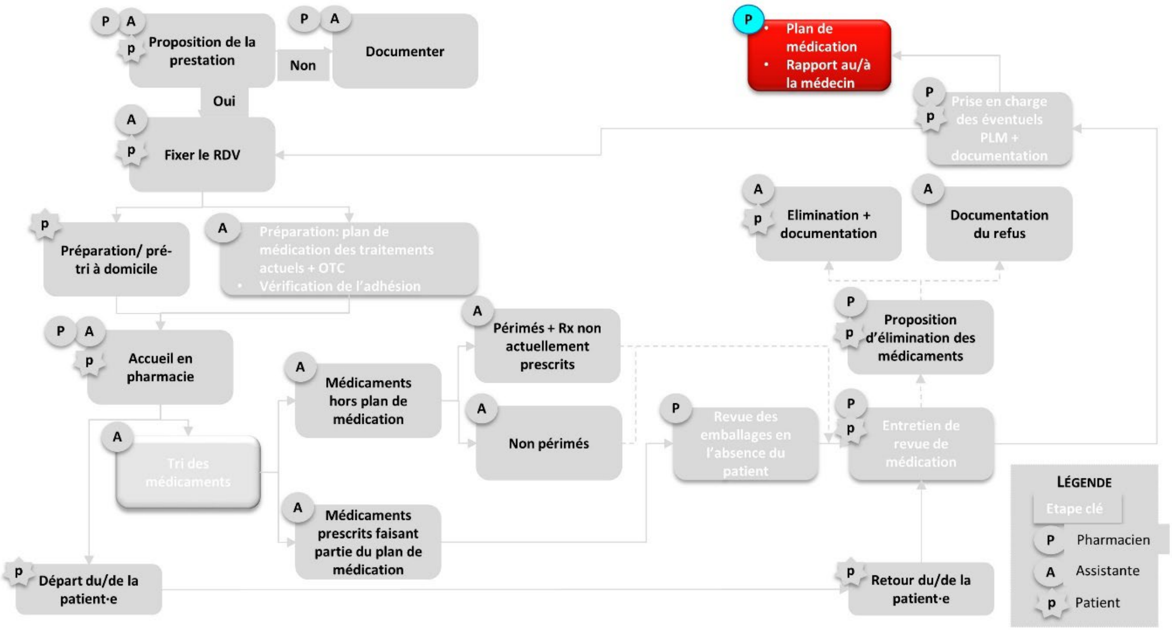

Après: Plan de médication actualisé et rapport au médecin

- **Qui** : Pharmacien·ne
- **But** : communication du rendu final de la prestation
  - Plan de médication actualisé = ce que prend réellement le/la patient·e. Si besoin à valider avec le/la prescripteur·trice
  - Bref rapport au/à la médecin (aide via des phrases types proposées selon le type de problèmes rencontrés)

Projet de recherche
Brève introduction

Objectifs principaux

- **Efficacité** de la prestation
- **Documentation** et **valorisation** des interventions pharmaceutiques
- Conséquences **économiques** des PLM
- **Satisfaction** des pharmacien·ne·s et des patient·e·s
- **Intérêt** et **faisabilité** de la prestation
- Description de la **fidélité** à la prestation

Projet de recherche: Principaux délais

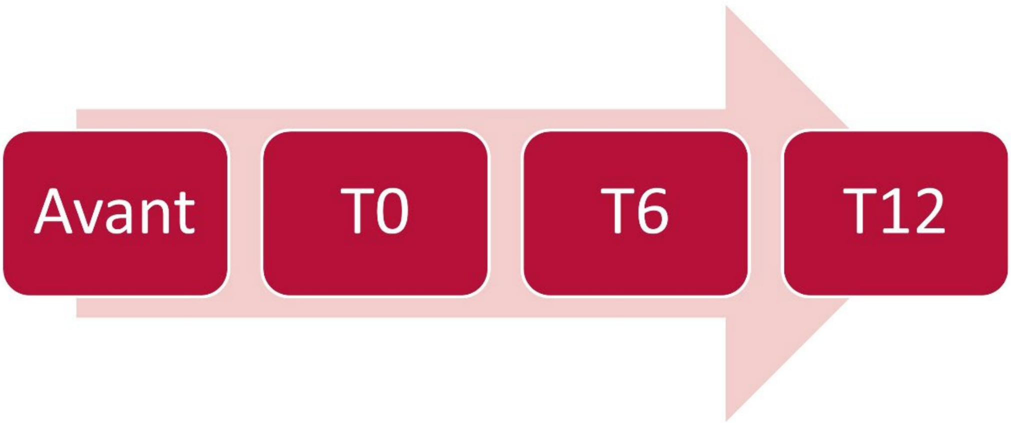

Avant
- **Pharmacien·ne·s**: recrutement, inclusion, formation
- **Patient·e·s**: recrutement et inclusion

T0
- 1er **entretien**
- **Questionnaire** de satisfaction
- **Envoi** des documents à l'équipe de recherche

T6
- 2ème **entretien**
- **Questionnaires** de satisfaction
- **Envoi** des documents à l'équipe de recherche

T12
- 3ème **entretien**
- **Questionnaires** de satisfaction
- **Envoi** des documents à l'équipe de recherche

Recrutement des patient·e·s

1. Les patient·e·s potentiellement admissibles à l'étude seront **identifié·e·s par la pharmacie**
2. Liste anonyme avec les patient·e·s admissibles transmise à l'équipe de recherche
3. Sélection aléatoire de 50 patient·e·s par l'équipe de recherche avec attribution d'un numéro patient·e → Diminution du **biais de sélection**
4. Environ **5-10 patient·e·s par pharmacie** (objectif de 162 patient·e·s à recruter dans 19-35 pharmacies)

Bonne pratique de remplissage des outils

Les outils de la prestation

- Outil composé de trois formulaires
  - «Revue de médication»
  - «Médicaments potentiellement à éliminer»
  - «Formulaire des Interventions pharmaceutiques: PharmDISC»

- Un plan de traitement actualisé

→ Les formulaires et le plan de traitement sont proposés sous forme électronique dans un unique fichier excel pour faciliter le remplissage des documents. Ils seront aussi disponibles sous forme papier

Appendix 2: Eligible patients form

Formulaire d’inclusion des patient.e.s

Identification du/de la patient·e		Critères d'inclusion		Critères d’exclusion							Sélection du/de la patient·e	Inclusion du/de la patient·e	Raison de refus exprimée par le/la patient·e
**Ordre de prise de contact**	Code patient·e	Prise d'au moins 4 médicaments chroniques depuis au moins 3 mois	Patient·e majeur·e	Problème de démence, désordre psychiatrique ou un autre problème de santé empêchant l’ obtention de son consentement éclairé et/ou la réalisation d’ un entretien avec le pharmacien	A déjà bénéficié d’ une réconciliation médicamenteuse dans les six mois précédent son entrée dans l’ étude	Ne parle pas couramment français	Incapacité de déplacer tous ses médicaments	Refuse de s’ entretenir avec le pharmacien dans T0, T6 et T12	Refuse de participer à l’ étude	Refuse la transmission du plan de médication et le rapport des PLM au médecin généraliste	Invité·e à participer à l’étude	Accepte de participer à létude	
**1**													
**2**													
**3**													
**4**													
**5**													
**6**													
**7**													
**8**													
**9**													
**10**													
………													
**45**													
**46**													
**47**													
**48**													
**49**													
**50**													

Appendix 3: Oral informed consent

Les patient.e.s devront donner leur consentement oral (le pharmacien.ne devra noter le consentement dans le formulaire d'inclusion des patient.e.s, Annexe 1) :

MAJ : Consentement des patient.e.s
Le/la patient.e accepte que ses données obtenues dans le cadre du projet « Médicaments à Jour ? » pour la revue de médicaments prise dans la pharmacie, soient transmises à son·sa médecin prescripteur·rice et puissent être réutilisées à des fins de recherche sous forme codée.

Il/elle sait que ses données sont conservées sous forme codée et que la liste d'identification est gardée dans un lieu sûr. Les données peuvent être envoyées à des fins d'analyse à une autre banque de données située en Suisse ou à l'étranger, à condition qu'elle obéisse à des normes et exigences au moins équivalentes aux normes et exigences suisses. Toutes les dispositions légales relatives à la protection des données sont respectées.

Le/la patient.e donne son accord de façon volontaire et il/elle sait qu'il/elle peut à tout moment revenir sur sa décision. S'il/elle revient sur sa décision, sans justification nécessaire, elle peut informer son/sa pharmacien.ne de contact, qui informera le groupe de recherche responsable du projet.

Appendix 4: Study flyer

Vous prenez plusieurs médicaments ? Votre armoire à pharmacie déborde et vous avez besoin de faire un tri dans ces médicaments ?

Votre pharmacien peut vous aider ! Sur rendez-vous, lors d'un entretien privé, vous pourrez discuter avec lui de vos médicaments, qu'ils soient prescrits ou en automédication et de leur gestion (prise, conservation, élimination, etc.). Deux autres rendez-vous seront fixés : à 6 mois et à 12 mois pour vous garantir un suivi optimal. Chaque entretien, d'une valeur de CHF 75.-, vous sera offert.

Comment ça marche ?

- Fixez un rendez-vous avec votre pharmacien (30 à 45 minutes).
- En vue de l'entretien, mettez dans un sac tous les médicaments de votre pharmacie de ménage, ainsi que ceux qui se trouveraient à un autre endroit (par exemple : cuisine, chambre, salle de bains). N'oubliez pas de placer les médicaments devant être réfrigérés dans l'enveloppe isolante qui vous a été remise. Vos médicaments vous seront restitués après l'entretien.

Après cette visite, vos médicaments vous seront rendus avec un plan de médication qui pourra également être transmis à votre médecin. Vous pourrez laisser à la pharmacie les médicaments dont vous n'aurez plus l'utilité.

Médicaments à apporter à l'entretien (qu'ils aient été achetés en pharmacie ou ailleurs) :

- Tous les comprimés, en blister ou dans leur carton (dans ou hors de leur emballage)
- Patchs (par ex. : patchs antidouleurs, hormonaux, contraceptifs, etc.)
- Gels, crèmes et lotions
- Liquides et solutions buvables
- Injectables (= seringues ou flacons, par ex. : insuline, etc.)
- Gouttes et pommades pour les yeux
- Sprays pour le nez ou la bouche et produits à inhaler (par ex. : pour soigner les crises d'asthme)
- Médicaments naturels, homéopathiques, vitamines et compléments alimentaires
- Suppositoires et ovules

Prendre tous les médicaments qu'ils soient intacts ou déjà ouverts, avec ou sans leur emballage, même périmés.

N'oubliez pas les produits que vous gardez au frigo !

Amenez également votre semainier ou autre dispositif destiné à vous aider dans la prise de vos médicaments (sachets, etc.)

Il n'est pas nécessaire d'amener les produits suivants :

- Pansements et matériel de soins (compresses, ciseaux, bandages, etc.)
- Thermomètres, tensiomètres, oxymètres ou autres appareils de mesure
- Aiguilles, seringues
- Cosmétiques, produits d'hygiène (shampoings, crèmes pour le corps, etc.)

Appendix 5: Medication management plan

Entretien à T 0 | Date entretien: 16.01.23 | N° Patient·e 0101

Médicaments prescrits			Dosage	Posologie					Nom du/de la médecin prescripteur·rice (A enlever avant envoi)	Utilisation des médicaments (scores -1=Incorrect ; 0=Ne sait pas ; 1=Correct ;								PLM/ Intervention pharmaceutique
DCI	Nom commercial	Forme galénique		Matin	Midi	Soir	Coucher	Réserve		Indication ?	Quand ?	Combien ?	Repas ?	Oublis ?	Effets indésirables ?	Conservation?	Score par médicaments	
																	0	
																	0	
																	0	
																	0	
																	0	
																	0	
																	0	
																	0	
																	0	
																	0	
																	0	
																	0	
																Score total :	0	
										Score total/nombre de médicaments :							#DIV/0!	

Médicaments non prescrits Nom commercial - forme galénique	Dosage	Mode de prise					Indication rapportée par le/la patient·e	PLM/ Intervention
Le/la patient·e a pris un traitement non prescrits durant les trois derniers mois? Non								

Remarques:

Version 04, 21 03 2023

Appendix 6: Removed medication plan

A compléter pour la recherche							
N° patient·e	0101	N° entretien (Tx)	T0	Visa Assistant·e (A)	A	Visa pharmacien·ne (P)	P
A compléter à T0 seulement	Catégorie d' âge	18 à 29 ans	Sexe	Homme	Niveau de formation		

Date de l'entretien	19.12.22					
Tâche	Préparation des documents	Tri des médicaments	Revue des emballages avant l'entretien	Entretien avec le/la patient·e	Rédaction du plan de médication	Rapport médecin
Durée (min + personne responsable A ou P)						
Décompte des emballages	Nombre d'emballages du groupe I:			Nombre de médicaments éliminés:		
	Nombre d'emballages du groupe II:					
Interventions	Non	Nombre d'intervention complétée:				

Un pré-tri des médicaments a été fait à domicile

Absence volontaire de posologie

Médicaments potentiellement à éliminier (DCI + Forme galénique)	Dosage	Problèmes liés aux médicaments relevés/suspectés			Médicament éliminé (O/N)
		Périmé	Ancien traitement	Autre problème	

Remarques:

Appendix 7: PharmDisc tool for drug related problems related to patients

1.1. MaJ ?- FORMULAIRE DES PLM/INTERVENTIONS PHARMACEUTIQUES

Numéro patient	

COMPLÉTER **UNE** PAGE PAR **P**ROBLÈME **(PLM),** REMPLISSEZ AUTANT DE PAGES QUE NÉCESSAIRES

Date : ________________ T0 ☒ T6 ☒ T12☐

Médicament(s) concerné(s) :____

STATUT DU PLM

☐ Nouveau
☐ Partiellement résolu/ Requiert un suivi dans (indiquer le temps):
☐ Ne sait pas

Un PLM peut requérir plusieurs interventions, cochez toutes les cases applicables proposées lors de sa détection

PharmDISC :Documentation d'interventions pharmaceutiques

Raison de l'intervention (1 choix)

1. Choix du traitement
☐ 1. Doublon
☐ 2. Accumulation
☐ 3. Effet indésirable

2. Choix de la dose
☐ 1. Sous-dosage
☐ 2. Surdosage

3. Utilisation du médicament
☐ 1. Moment / fréquence d'admin. inappropriée
☐ 2. Modalité d'utilisation inappropriée
☐ 3. Durée du traitement inappropriée

4. Patient
☐ 1. Mauvaise adhésion du patient
☐ 2. Préoccupations concernant le traitement
☐ 3. Charge financière (Patient / syst. santé publique)
☐ 4. Stockage inapproprié
☐ 5. Etiquette absente

5. Logistique
☐ 1. Médicament prescrit non disponible
☐ 2. Erreur dans le circuit du médicament

6. Autre
☐ 1.……………………………………………………………………

Intervention

☐ 1. Substitution / échange
☐ 2. Adaptation posologique
☐ 3. Adaptation taille/nombre d'emballages
☐ 4. Optimisation des modalités / voie d'administration
☐ 5. Arrêt d'un traitement/ pas de remise
☐ 6. Initiation/ continuation d'un traitement
☐ 7. Conseil approfondi au patient
☐ 8. Instruction de l'utilisation (éducation)
☐ 9. Remise d'une aide à l'adhésion, conseil inclus
☐ 10. Clarification / ajout d'informations
☐ 11. Transmission d'informations
☐ 12. Proposition d'un suivi thérapeutique
☐ 13. Autre : …………………………………………………………

Communication: personnes impliquées outre pharmacien

☐ 1. Aucune
☐ 2. Médecin
☐ 3. Soignant / soins à domicile
☐ 4. Patient / parent

Devenir de l'intervention

☐ 1.a. Acceptée et modifiée par le patient
☐ 1.b. Acceptée et modifiée par le prescripteur
☐ 2. Partiellement acceptée ou acceptée sans modification
☐ 3. Non accepté
☐ 4. Suivi inconnu
☐ 5. Non applicable

Remarques :

Appendix 8: Updated medication plan

Médicaments prescrits			Dosage	Posologie					Nom du/de la prescripteur·trice	Remarques
DCI	Nom commercial	Forme galénique		Matin	Midi	Soir	Coucher	Réserve		

Médicaments non prescrits<br>Nom commercial - forme galénique	Dosage	Mode de prise					Indication rapportée par le/la patient·e	Remarques
**Aucun médicament non prescrit n'a été pris pendant les trois derniers mois**								

Remarque : ce plan de médication ne remplace pas tout nouvel ordre médical. Il décrit les médicaments pris par le/la patient·e sur prescription tout comme l'automédication utilisée durant les 3 derniers mois selon un entretien avec le/la pharmacien·ne.

Appendix 9: Satisfaction questionnaire for pharmacists

Numéro d'identification	

MÉDICAMENTS À JOUR? (MAJ)
QUESTIONNAIRE SUR LA SATISFACTION ENVERS LA PRESTATION
À L'INTENTION DES PHARMACIEN.NE.S

Ce formulaire a pour but d'évaluer votre niveau de satisfaction concernant la formation vous qui a été offerte et l'outil que vous avez utilisé durant l'étude. Veuillez cocher la réponse correspondant le mieux à votre niveau de satisfaction pour chacun des éléments suivants

**Votre opinion** / **Éléments évalués**	Tout à fait d'accord	D'accord	Indifférent	Pas d'accord	Pas du tout d'accord
Le contenu de la formation est pertinent pour ma pratique					
La prestation MAJ est pertinente pour ma pratique					
Les documents liés à MAJ me permettent de collecter facilement les informations nécessaires à la gestion des PLM.					
Les documents liés à MAJ me permettent de collecter plus facilement les informations nécessaires pour une bonne communication avec le patient.					
Je n'ai pas eu de problèmes pour proposer cette prestation à mes patient.e.s					
La prestation MAJ m'a permis de collaborer plus souvent avec des médecins					
La prestation MAJ m'a permis de communiquer plus souvent avec les patient.e.s					
La prestation MAJ me permet de renforcer ma place dans le réseau interprofessionnel autour des patient.e.s					

Quels aspects de la prestation MAJ avez-vous appréciés?

____
____
____
____

Quels aspects de la prestation MAJ seraient à améliorer?

Quelle est votre évaluation globale :
De la formation?

De l’approche MAJ (réconciliation en présence de tous les emballages) ?

Des documents utilisés lors de la prestation MAJ?

Avez-vous d’autres commentaires?

Nous vous remercions votre précieuse collaboration!
