# Supplementary material for: Evaluation of an enhanced service for medication review with follow up in Swiss community pharmacies: Pre-post study protocol
Source: PLoS One. 2023 Oct 17;18(10):e0292037. doi: 10.1371/journal.pone.0292037 (PMC10581489; doi:10.1371/journal.pone.0292037)
Supplement: S6 Appendix — (PDF) [file pone.0292037.s006.pdf]

**Sponsor / Sponsor-Investigator**	*PD Dr Jérôme Berger, Pharmacien chef, Centre universitaire de médecine générale et santé publique, Unisanté, Secteur Pharmacie – Recherche. Rue du Bugnon 44, 1011 Lausanne, Suisse / Institut des Sciences Pharmaceutiques de Suisse Occidentale, Université de Genève, Université de Lausanne. Rue Michel Servet 1, 1206 Genève, Suisse. 021 314 48 43 (secrétariat). jerome.berger@unisante.ch.*
**Study Title:**	*Médicaments à Jour? - MaJ? Study of feasibility of a pharmaceutical service for patients with polypharmacy*
**Short Title / Study ID:**	*Médicaments à Jour ? – MaJ?*
**Protocol Version and Date:**	*Version 4, 21.03.2023*
**Trial registration:**	*Clinicaltrials.gov NCT05348538*
**Study category and Rationale**	*Category A as there is no sampling for biological material and the burdens or risks while collecting data is minimal for the patients. The interviews carried out by the pharmacists with patients do not present risks to patients and allows to structure and document clinical activities usually conducted in community pharmacies.*
**Background and Rationale:**	*In Switzerland, 20,000 people are hospitalized each year as a result of drug related problems (DRP). There are many sources of DRP, such as incorrect storage or missing dosages from packages. Community pharmacists are well positioned to identify these DRPs related to patients and manage them in a timely manner.*<br>*The Société Vaudoise de Pharmacie (SVPh) and Unisanté have developed a new service, "Médicaments à Jour? (MaJ?)". The patient brings all medications to the community pharmacy, where the pharmacist reviews them. A medication review between the pharmacy record and the medication brought by the patient is performed. Pharmacists also communicate the medication plan and the DRPs detected to the patient and their general medical practitioner. In addition, at the moment of the pharmacist-patient consultation, the patient is given the option to leave their expired or untaken medications for appropriate disposal.*
**Objective(s):**	*Primary objective:*<br>*To evaluate the impact of MaJ? on the identification and management of DRP.*<br>*Secondary objectives:*<br>*To assess the impact of MaJ? on the number of expired or untaken medications.*<br>*To assess the impact of MaJ? on patients' knowledge about their treatments.*<br>*To describe the interventions made by the pharmacists through MaJ?.*
**Outcome(s):**	*Numbers of DRPs detected, their classification (PharmDISC tool) and evolution.*<br>*Number of medications removed*<br>*Patients knowledge about their medication*<br>*Number of pharmaceutical interventions and their classification (PharmDISC tool)*<br>*Service duration*<br>*Participation rate*
**Study design:**	*Pre-post intervention study carried out in Swiss community pharmacies from the canton of Vaud (multicenter).*

**Inclusion / Exclusion criteria:**	*In order to participate in this study, patients must meet the following inclusion criteria:*<br>*- Patients with a prescription for at least four chronic drugs for at least the last three months.*<br>*- Adult patients, 18 years old or older.*<br>*Patients meeting one or more of the following exclusion criteria will not be included in the study:*<br>*- Patients suffering from dementia, psychiatric disorder, or other health condition that hinders obtaining informed consent and/or conducting the consultations with the pharmacist.*<br>*- Patients receiving a PMC within the last six months prior their study enrollment.*<br>*- Patients who disagree meeting the pharmacist for the first consultation (T0), second consultation six months later (T6) or third consultation twelve months after the first one (T12).*<br>*- Patients who are not able to bring all their medication to the pharmacy.*<br>*- Patients who cannot speak and read French.*<br>*- Patients who does not allow the pharmacist contacting the general medical practitioner to inform him/her about possible DRPs.*<br>*- Patients who will not consent of participating in the study.*
**Measurements and procedures:**	*The pharmacists will carry out three different consultations with each patient who agrees to participate, at the beginning of the study (T0), six months later (T6) and 12 months after the first encounter (T12).*
**Study Product / Intervention:**	*The pharmacist will check the patient's medication to identify potential DRPs and propose interventions to solve them. The information evaluated by the pharmacist will be based on the pharmacy record, treatments brought by the patient to the pharmacy and the information obtained during the consultation with the patient.*<br>*The service will be provided to the patient in three moments (beginning of the study, six months later and twelve months later) to evaluate the evolution of possible DRPs and patient's knowledge.*
**Control Intervention (if applicable):**	*NA*
**Number of Participants with Rationale:**	*Sample size calculation is based on the primary outcome to detect a difference of 0.5 DRP per patient at the end of the study. The sample size was calculated with ≥0.8 power, type I error rate of 5%, assuming an intra-cluster correlation of 0.02. Allowing for 15% dropout, the overall sample size is 162 patients, with 19 to 35 pharmacies (1-10 patients per pharmacy).*
**Study Duration:**	*The study will be carried out for 15 months, from Avril 2023 until June 2024.*
**Study Schedule:**	*Month Year of First-Participant-In (planned) : April 2023*<br>*Month Year of Last-Participant-Out (planned) : May 2024*
**Investigator(s):**	*Community pharmacists will be involved at the beginning of the study. Names, addresses and signatures will be sent to the Ethics Committee throughout the study.*
**Study Centre(s):**	*Multi centre study, between 19 to 35 community pharmacies in the canton of Vaud will be enrolled at the beginning of the study.*

**Statistical Considerations:**	*Continuous variables will be reported using mean and standard deviation, or median and percentiles depending on the distribution of the variable. Categorical variables will be reported using frequency and proportion. For the comparison of continuous variables, the T Student's test or the ANOVA test will be carried out if there is a normal distribution, and Kruskal-Wallis otherwise. The comparison of the categorical variables will be carried out using the χ2 test, the Fisher's exact test or Yate's chi-squared test if necessary.*<br><br>*For each patient under study, differences T12-T0 and T6-T0 will be compared for dependent variables (DRP, pharmaceutical interventions, patient knowledge, and medication removed). A linear regression model will be performed accounting for the cluster effect. Firstly, variables will be considered significant (p-value<0.2) in a bivariate model to be included in a multivariate model. Secondly, the variables considered significant at a value of p<0.1 in the multivariate model will be included in the final model.*
**GCP Statement:**	*This research project will be conducted in accordance with the protocol, the Declaration of Helsinki (46), the principles of Good Clinical Practice, the Human Research Act (HRA) and the Human Research Ordinance (HRO)as well as other locally relevant regulations.*
